# Basidiomycota Fungi and ROS: Genomic Perspective on Key Enzymes Involved in Generation and Mitigation of Reactive Oxygen Species

**DOI:** 10.3389/ffunb.2022.837605

**Published:** 2022-03-23

**Authors:** Hans Mattila, Janina Österman-Udd, Tuulia Mali, Taina Lundell

**Affiliations:** Department of Microbiology, Faculty of Agriculture and Forestry, Viikki Campus, University of Helsinki, Helsinki, Finland

**Keywords:** reactive oxygen species (ROS), Basidiomycota, superoxide dismutase, catalase (CAT), thioredoxin (TRX) family proteins, NADPH oxidase (NOX), GMC oxidoreductases, CAZy AA auxiliary enzymes

## Abstract

Our review includes a genomic survey of a multitude of reactive oxygen species (ROS) related intra- and extracellular enzymes and proteins among fungi of Basidiomycota, following their taxonomic classification within the systematic classes and orders, and focusing on different fungal lifestyles (saprobic, symbiotic, pathogenic). Intra- and extracellular ROS metabolism-involved enzymes (49 different protein families, summing 4170 protein models) were searched as protein encoding genes among 63 genomes selected according to current taxonomy. Extracellular and intracellular ROS metabolism and mechanisms in Basidiomycota are illustrated in detail. In brief, it may be concluded that differences between the set of extracellular enzymes activated by ROS, especially by H_2_O_2_, and involved in generation of H_2_O_2_, follow the differences in fungal lifestyles. The wood and plant biomass degrading white-rot fungi and the litter-decomposing species of Agaricomycetes contain the highest counts for genes encoding various extracellular peroxidases, mono- and peroxygenases, and oxidases. These findings further confirm the necessity of the multigene families of various extracellular oxidoreductases for efficient and complete degradation of wood lignocelluloses by fungi. High variations in the sizes of the extracellular ROS-involved gene families were found, however, among species with mycorrhizal symbiotic lifestyle. In addition, there are some differences among the sets of intracellular thiol-mediation involving proteins, and existence of enzyme mechanisms for quenching of intracellular H_2_O_2_ and ROS. In animal- and plant-pathogenic species, extracellular ROS enzymes are absent or rare. In these fungi, intracellular peroxidases are seemingly in minor role than in the independent saprobic, filamentous species of Basidiomycota. Noteworthy is that our genomic survey and review of the literature point to that there are differences both in generation of extracellular ROS as well as in mechanisms of response to oxidative stress and mitigation of ROS between fungi of Basidiomycota and Ascomycota.

## Introduction

Organisms living in aerobic or anaerobic environments encounter oxidative stress both resulting from their cellular metabolism and activities of other organisms (biotic stress) as well as due to environmental conditions (abiotic stress). The detrimental effects of oxidative stress are attenuated by conserved cellular sensing mechanisms, and multiple antioxidant enzymes and proteins essential for all living organisms. Oxidative stress may be a consequence and organismal response to different abiotic factors, such as changes in temperature and moisture conditions, light and radiation, presence of harmful chemicals and metals, and deprivation of nutrients. In addition, pathogen-host and microbe-microbe interactions create oxidative stress responses in the susceptible partners and in microbial consortia of various environments.

With all this in mind, it is obvious that biological quenching mechanisms against oxidative stress are essential for life. Cellular oxidative stress is universal to all organisms and in eukaryotes, oxidative reactions compartmentalize in the cellular organelles (mitochondria, chloroplasts, peroxisomes, e.g.). Together with tolerance against oxidative stress, living organisms actively produce intracellular and extracellular oxidative agents for essential cellular pathways and metabolic activities.

### Fungi and Oxidative Stress

In this review study, we give an overview of enzymes involved in production and mitigation of oxidative agents performed in the fungi of *Basidiomycota* which represent diverse lifestyles (independent saprobic, symbiotic, or pathogenic). Focus is on the impact of formation, quenching and utilization of the most ubiquitous oxidative agents, the reactive oxygen species (ROS). In cell biology, oxidative agents like ROS are associated with harmful effects (Packer and Cadenas, 2007). However, ROS and reactive nitrogen species (RNS) are also important signaling molecules and regulators in cellular development and metabolism both in microbes and multicellular eukaryotes (D'Autreaux and Toledano, 2007; Sies and Jones, 2020).

In fungi, these reactive compounds and radicals (ROS and RNS) are essential to sustain core metabolism and intracellular activities. Considering production of fungal extracellular secondary metabolites (SM) with antioxidant activity, environmental stress and excess of ROS are known factors promoting expression of SM encoding biosynthetic gene clusters (Brakhage, 2013; Keller, 2019). Furthermore, ROS are important for fungal extracellular redox reactions involving enzymes and radical chemistry (Bissaro et al., 2018). The extracellular oxidative reactions enable fungi to decompose complex substrates like plant biomass to colonize new habitats and are involved in pathogen-host interactions.

Within the next chapters, we discuss the primary mechanisms and enzymes that generate, utilize or diminish ROS (and RNS) in fungi of the phylum *Basidiomycota*. Previously, the role of ROS in plant-pathogenic fungi has been reviewed (Heller and Tudzynski, 2011), and mitigation of oxidative stress in a selection of *Ascomycota* species was summarized with a focus on intracellular enzymes (Breitenbach et al., 2015). However, we emphasize that an expansion of the survey among fungi is needed, for instance considering additional lifestyles (like decay of wood) which are uniquely found among *Basidiomycota*. For this purpose, we performed a bioinformatic comparative genomic survey of 49 different ROS and RNS related enzyme-encoding gene families among over 60 species of genome-sequenced *Basidiomycota* (Supplementary Information: Materials and methods). Fungal species were selected according to coverage of taxonomic orders and lifestyles (Supplementary Table 2). We analyzed 4170 protein models (Supplementary Table 1), also with prediction of localization, and inspected each protein family by comparison to known reference protein sequences.

### Reactive Species and Radicals in Oxidoreductive Reactions and Responses

Biological, intracellular oxidative agents are in principal either reactive oxygen species (ROS) or reactive nitrogen species (RNS) (Fang, 2004). Typical intracellular ROS are: (1) superoxide (${\text{O}}_{2}^{•-}$) which is generated from molecular oxygen (O_2_) accepting an additional electron, (2) hydrogen peroxide (H_2_O_2_) which is formed by two-electron reduction of molecular oxygen or reduction of superoxide by the enzyme superoxide dismutase (SOD), and (3) highly reactive hydroxyl radicals (HO^•^), which may be generated through Fenton chemistry, decomposition of hydroperoxides, or by photochemical reactions (D'Autreaux and Toledano, 2007; Prousek, 2007; Herb et al., 2021). Additional intracellular ROS are (4) hydroperoxyls, peroxyl and alkoxyl radicals generated in lipid peroxidation reactions (Ayala et al., 2014; Halliwell and Gutteridge, 2015).

The origin of reactive nitrogen species (RNS) is usually nitric oxide (•NO, nitrogen oxide), which causes cellular nitrosative stress when reacting with ROS compounds, usually with superoxide (del Río, 2015; Di Meo et al., 2016). Nitrosative RNS comprise peroxynitrite (ONOO^−^), nitrogen dioxide (•NO_2_), and dinitrogen trioxide (N_2_O_3_). ROS separately and together with nitrosative agents may in turn cause lipid peroxidation in cell and organelle membranes, oxidation and nitration of proteins, DNA damage and even initiation of apoptosis (Di Meo et al., 2016). Formation of RNS and ROS are metabolically connected by intracellular enzymes which generate •NO and ${\text{O}}_{2}^{•-}$ and will be discussed below in Part II.

## Part I: Extracellular ROS

### Extracellular ROS in *Basidiomycota*

Many vital functions in fungi are dependent on sufficient input of oxidative agents. Furthermore, animal or plant pathogenic organisms encounter hydrogen peroxide and superoxide in their environments, which may be enzymatically controlled, and sensed by specific transmembrane sensor and receptor proteins functional in recognition of oxidative stress (Packer and Cadenas, 2007). Thereby, extracellular ROS may trigger specific cellular metabolic and genetic responses in fungi.

**Figure 1:**
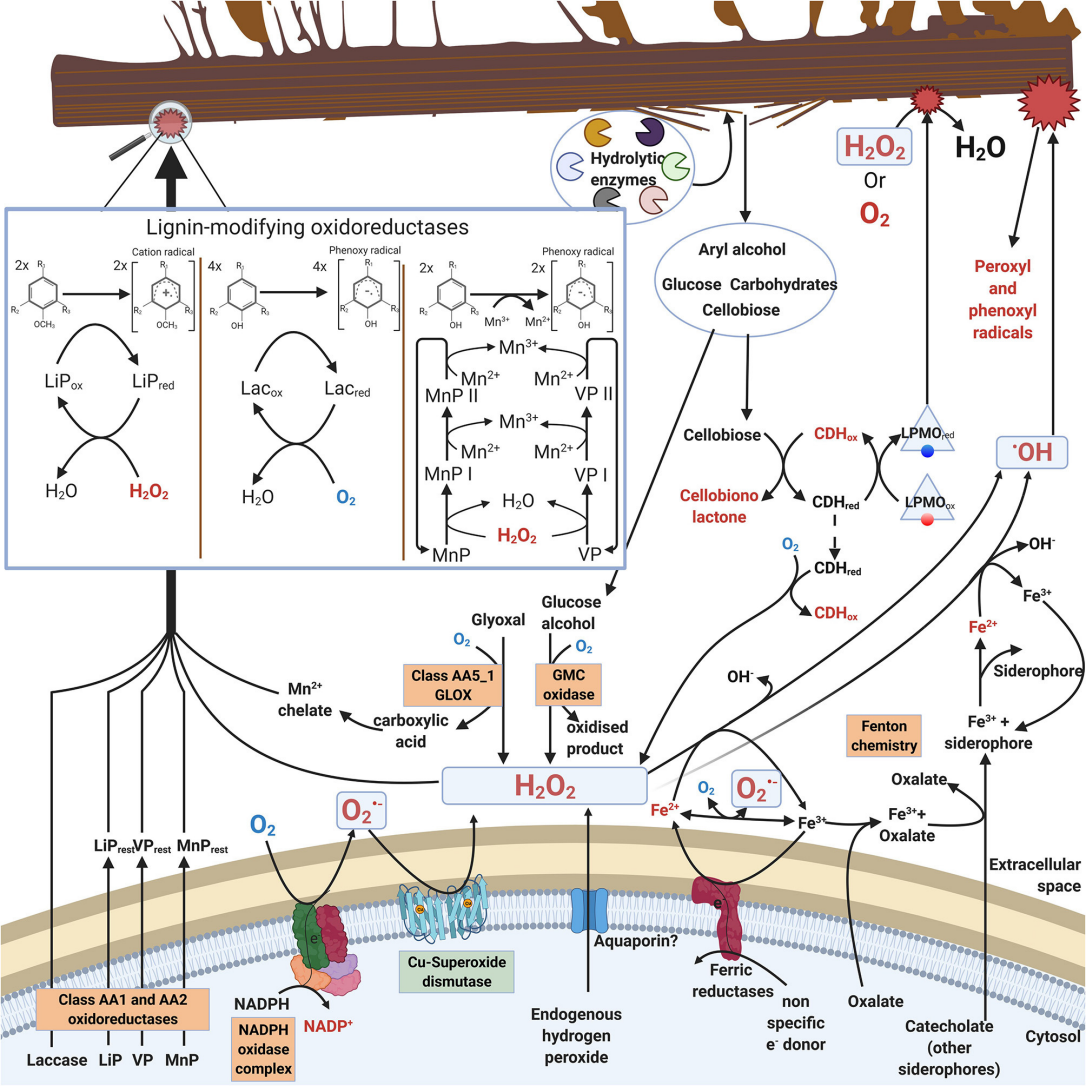
Extracellular ROS formation in fungi of Basidiomycota illustrating reactions and enzymes involved. For clarity, stoichiometry is not included in the catalytic reactions. Boxed enzymes are discussed in the text.

In saprobic (saprotrophic) fungi, enzymatic decomposition of plant biomass and wood lignocellulose is dependent on secreted enzymes including various oxidoreductases. In this case, ROS (mainly H_2_O_2_) are needed as oxidative (first) substrate molecules to initiate enzyme catalysis (Hofrichter et al., 2010; Lundell et al., 2010; Bissaro et al., 2018). Additionally, ROS are involved in extracellular Fenton chemistry which is specific for brown rot fungal decay of wood (Hatakka and Hammel, 2010; Arantes and Goodell, 2014; Lundell et al., 2014). Enzymatic reactions for production of extracellular ROS in filamentous saprobic *Basidiomycota* fungi are illustrated in Figure 1.

Saprobic fungi are dependent on extracellular degradation of plant biomass and organic biopolymers for their sources of energy, carbon, and nitrogen. For this purpose, these fungi secrete carbohydrate-active extracellular enzymes, CAZymes (Lombard et al., 2014; http://www.cazy.org/). The array of CAZymes include specific hydrolytic enzymes (cellulases, hemicellulases, pectinases, esterases, chitinases) as well as electron-transferring auxiliary oxidoreductases (laccases, oxidases, peroxidases, peroxygenases). The auxiliary oxidoreductases either produce or require ROS in their catalytic reactions (Figure 1, Table 1, chapters 2.1 and 2.2).

**Table 1:** Extracellular ROS enzymes and reactions in *Basidiomycota*.

**Protein superfamily**	**InterPro domain classification**	**CAZy classification**	**Prosthetic group or cofactor**	**EC class-ification**	**Secreted enzymes**	**UniProt examples**	**Related non-secreted enzymes**	**Oxidant**	**1st Product**	**Reductant**	**2nd Product**	**Functions for fungi**	**References**
**ROS producing oxidoreductases**													
GMC (glucose-methanol-choline)	IPR012132	AA3	FAD	1.1.3.4, 1.1.3.7, 1.1.3.12, 1.1.3.13, 1.1.99.18, 1.1.99.29	Aryl-alcohol oxidase, Pyranose dehydrogenase, Glucose oxidase—glucose dehydrogenase, Cellobiose dehydrogenase	D3YBH4, P56216, Q3L245, Q01738, Q3L1D1	Alcohol (methanol) oxidase, Pyranose oxidase	O_2_	H_2_O_2_	Aryl-alcohol, glucose, cellobiose, oligosaccharides, methanol	Aryl-aldehyde, glucono-1,5-lactone, cellobiono-1,5-lactone	Production of extracellular H_2_O_2_, electron transfer to other oxidoreductases	Sützl et al., 2019; Karppi et al., 2020
CRO (mononuclear copper-radical oxidase)	IPR009880	AA5	Cu atom	1.1.3.9	Glyoxal oxidase, Galactose oxidase	Q01772, P0CS93		O_2_	H_2_O_2_	Glyoxal, glycerol, monosaccharides	Glyoxylic acid, pyranose aldehydes	Production of extracellular H_2_O_2_	Yin D. et al., 2015; Daou and Faulds, 2017
NADPH oxidases*	IPR029650, IPR013121		2 Haems and FAD	1.6.3.1	Membrane bound, non-secreted multisubunit enzyme	A6ZIB7, A8P0A9, B0CPG0,	NOX, DUOX, Ferric reductase	O_2_	${\text{O}}_{2}^{𢀢}$	NADPH	NADP^+^	Primarily intracellular and organellar, ROS production for signaling and defense, involved in fungal pathogenesis	Takemoto et al., 2007; Panday et al., 2015; Magnani et al., 2017
**Not producing ROS but reducing O**_**2**_													
MCO (multicopper oxidase)	IPR017761 (others)	AA1	4 Cu atoms	1.10.3.2	Laccase, laccase-like multicopper oxidase	Q12718, Q70KY3,	Multicopper oxidase, ferroxidase, L-ascorbate oxidase	O_2_	2 H_2_O	Phenols, aromatic polymers,	Phenoxy radicals, quinones, coupled oligomers	Oxidation, polymerization and activation of phenolic compounds	Thurston, 1994; Hildén et al., 2009; Lundell et al., 2010
**ROS activated oxidoreductases**													
Haem peroxidase	IPR010255	AA2	Haem (Fe protoporphyrin IX)	1.11.1.13, 1.11.1.14, 1.11.1.16	Lignin peroxidase, manganese peroxidase, versatile peroxidase, low-redox peroxidase	P06181, Q02567, O94753, P28314	Cytochrome-c peroxidase, ascorbate-like peroxidase	H_2_O_2_	2 H_2_O	Aryl-alcohols, phenols, polymeric aromatic compounds, Mn^2+^	Cation radicals, phenoxy radicals, aromatic aldehydes and ketones, Mn^3+^ ions	Oxidation and degradation of lignins and polyphenols	Ruiz-Dueñas and Martínez, 2009; Hofrichter et al., 2010; Lundell et al., 2010
DyP-type peroxidase	IPR006314		Haem (Fe protoporphyrin IX)	1.11.1.19	Dye-decolorizing peroxidase	Q8WZK8, I2DBY1	Ferredoxins	H_2_O_2_	2 H_2_O	Polymeric dye molecules, anthraquinones	Quinones, ketones, aldehydes	Oxidation and bleaching of polymeric soluble dye molecules	Sugano and Yoshida, 2021
Chloroperoxidase-like superfamily	IPR036851		Haem (Fe protoporphyrin IX)	1.11.2.1	Heme-thiolate peroxidase, unspecific peroxygenase, aromatic peroxygenase	B9W4V6	CYP P450 enzymes	H_2_O_2_	ROH + H_2_O	H_2_O_2_ or aromatic compound	O atom inserted product, N-oxidized, Br-added products, radicals	Unspecific peroxidase, extracellular P-450 enzyme, mono-oxygenase	Pecyna et al., 2009; Hofrichter et al., 2010; Faiza et al., 2019
LPMO	IPR005103	AA9 (GH61), AA14,	Cu atom	1.14.99.54, 1.14.99.56	Lytic cellulose monooxygenase (C1- and C4-dehydrogenating activities), lytic polysaccharide monooxygenase, glycoside hydrolase family 61	Q8WZQ2, H1AE14, A0A2I6QAZ5	Non-degradative similar proteins secreted by fungi	H_2_O_2_ or O_2_	ROH + H_2_O	Cellulose, hemicellulose, pectin, chitin,	Cleavage products of the β-1,4 glycosidic oligomers	Degradation of plant polysaccharides and chitin	Bissaro et al., 2018, 2020; Tandrup et al., 2018
**Non-enzymatic use and production of ROS**													
Fenton chemistry								H_2_O_2_	HO^•−^			Oxidation, radical formation, degradation of plant polysaccharides and biomolecules	Jensen et al., 2001; Arantes and Goodell, 2014

During decomposition of plant biomass, saprobic fungi may then experience continuous exogenous oxidative stress caused by ROS (hydrogen peroxide or hydroxyl radical) together with the various organic radicals generated in oxidative degradation of the lignocellulose biopolymers (Figure 1). Hydrogen peroxide is an essential co-substrate in lignocellulose degradation, since the extracellular peroxidases and peroxygenases are activated by H_2_O_2_ (Hofrichter et al., 2010; Linde et al., 2015) (chapter 2.2). In extracellular redox enzyme-coupled reactions, hydrogen peroxide is generated by auxiliary oxidases (chapter 2.1) which are enzymes reducing O_2_ to H_2_O_2_ (Martínez et al., 2009; Kersten and Cullen, 2014; Lundell et al., 2014; Bissaro et al., 2018; Sützl et al., 2018). These extracellular oxidases represent several protein superfamilies (Table 1, chapter 2.1).

#### Extracellular ROS Producing Enzymes

##### NADPH Oxidases

NADPH oxidase is a membrane-embedded redox enzyme found in all eukaryotic lineages including animals, plants, fungi, and microbial eukaryotes (Kawahara et al., 2007). The NADPH oxidases (NOX) and related dual oxidases (Duox) are enzymes producing superoxide ${\text{O}}_{2}^{-\cdot }$ by reduction of O_2_. For NOX (EC 1.6.3.1), NADPH is the second, enzyme reducing substrate (Table 1). Human NOX are well-characterized, and their role in immune defense, phagocyte ROS formation, signaling and diseases has been elucidated in numerous studies (reviewed in Schröder, 2020; Vermot et al., 2021). Fungi have at least three different subfamilies of NADPH oxidases: NoxA, NoxB, and NoxC (Takemoto et al., 2007), and their role in ROS formation can range from cellular differentiation and signaling to defense and pathogenic fungus-host interactions (Egan et al., 2007).

NOX is a multidomain enzyme containing six transmembrane helices, and cytosolic domains binding flavin adenine dinucleotide (FAD) and the reducing substrate NADPH (Takemoto et al., 2007; Magnani et al., 2017). In catalysis, molecular oxygen O_2_ is bound to a specific cavity on the extracytoplasmic side in the protein complex, and linear arrangement of redox cofactors (NADPH, FAD, and two membrane-embedded heme moieties) transfers electrons from the intracellular side across the membrane to the oxygen-binding site (Magnani et al., 2017) (Figure 1). NOX activity is regulated from the cytosolic side by binding of the NoxR protein and a small GTP-binding protein, RacA, together with other components (Takemoto et al., 2007). Fungal NoxA is a typical NADPH oxidase similar in structure to human gp91^*phox*^ (Lara-Ortiz et al., 2003) while subfamilies NoxB and NoxC have additional N-terminal extensions (Takemoto et al., 2007).

**Figure 2:**
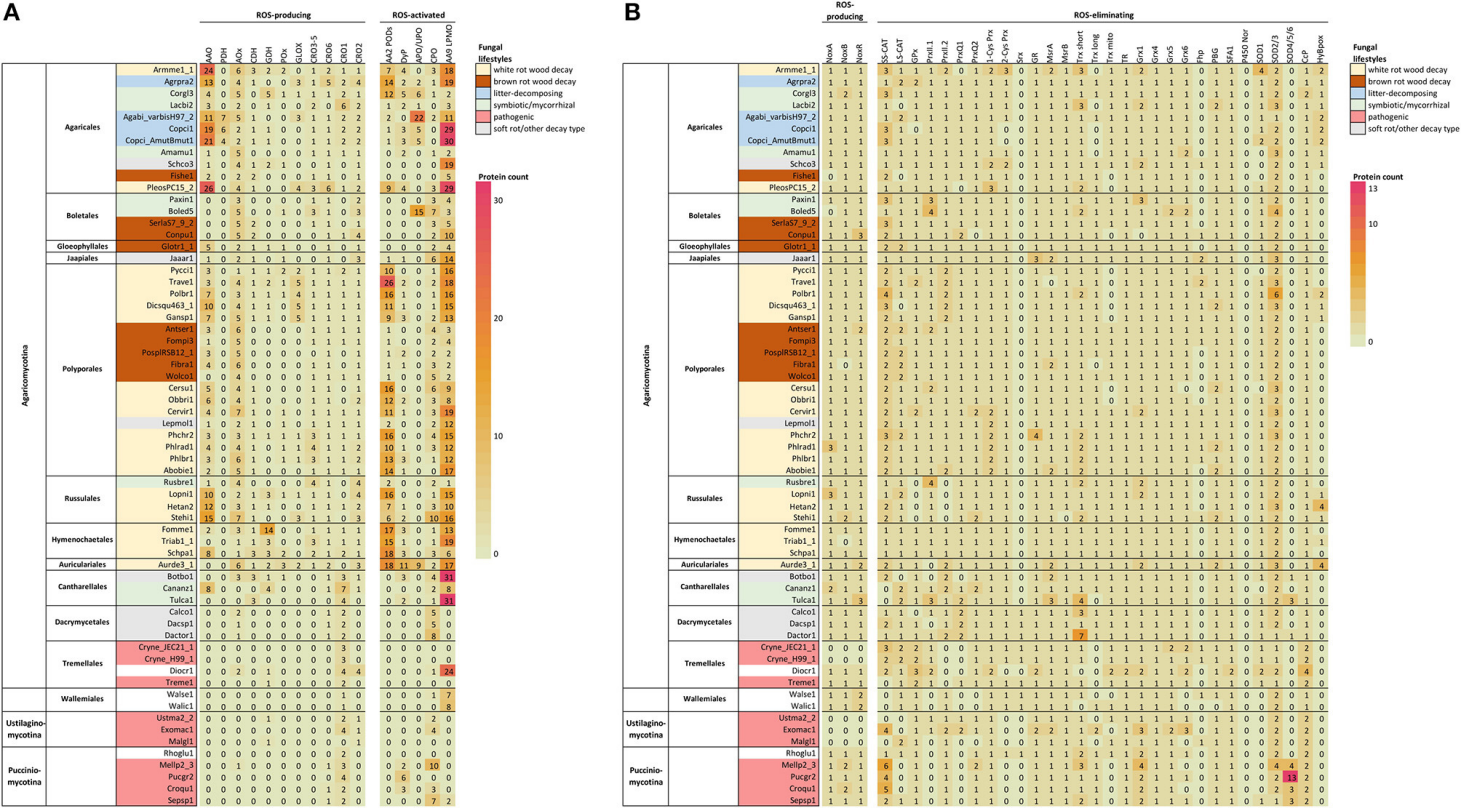
ROS enzymes in *Basidiomycota* according to taxonomy. **(A)** Extracellular enzyme, **(B)** intracellular enzyme and protein encoding genes involved in ROS generation, utilization or quenching reactions. Fungal lifestyles are depicted in different colors (legend on the top right corner). Counts represent protein models in each genome available at JGI MycoCosm (Supplementary Table 1). Genome & species coding: opened in Supplementary Table 2.

In previous studies, no correlation was found between fungal lifestyle and the set of genes coding for the different NOX isoforms and subunits (Grissa et al., 2010). Since then, availability of fungal genomes has greatly expanded both in number and taxonomic diversity. In our comparative search analysis, most of the selected species of *Basidiomycota* possessed in their genomes a single NoxA and one NoxB encoding gene together with a gene coding for the NoxR regulator (Figure 2B, Supplementary Table 1).

In our analysis, no NoxC encoding genes were found in any of the *Basidiomycota* genomes studied (Supplementary Table 1). Genes encoding proteins of NoxC type have been annotated in filamentous *Ascomycota* (*Fusarium graminearum, Magnaporthe grisea*) and in non-fungal eukaryotes like oomycetes and slime molds (Takemoto et al., 2007). Moreover, the genomes of *Cryptococcus neoformans* (class *Tremellomycetes*) and all members of subphylum *Ustilaginomycotina* were lacking genes encoding NoxA, NoxB, NoxC and NoxR (Figure 2, Supplementary Table 1), which is consistent with previous studies (Takemoto et al., 2007). *C. neoformans* and a majority of the species of *Ustilaginomycotina* are pathogenic to animals or plants. This pinpoints either their inability to generate extracellular ROS due to lack of a functional NOX enzyme, or presence of alternative enzymes and mechanisms for production of ROS in their pathosystems.

Most of the *Basidiomycota* NoxA homologs showed putative localization into the endoplasmic reticulum (ER) according to the DeepLoc analysis (Supplementary Table 1). NOX enzyme proteins are directed into the ER for maturation, but it remains unclear if, for instance, attachment of flavin and heme co-factors occurs in the ER or later in the cytosol. In other words, predicted localization into the ER may indicate processing of an immature or inactive enzyme. In contrast to NoxA localization, a majority of *Basidiomycota* NoxB homologs were predicted to become integrated into the cell membrane. NoxB is often associated with pathogenicity and its mammalian homolog Nox2 is functional in phagocytosis (Takemoto et al., 2007; Magnani et al., 2017; Schröder, 2020).

When coupled with superoxide dismutase (SOD) activity, NADPH oxidase may function as a source of hydrogen peroxide (Table 1). In saprobic *Basidiomycota* and *Ascomycota*, membrane-bound NOX could initiate production of extracellular H_2_O_2_ thus facilitating enzymatic and non-enzymatic decomposition of plant biomass (Figure 1). Production of extracellular H_2_O_2_ can be mediated by coupled activity of NADPH oxidase and a specific membrane-bound superoxide dismutase (SOD) (chapter 3.1.1).

##### GMC Oxidoreductases

The glucose–methanol–choline (GMC) superfamily of oxidoreductases, also known as the AA3 family in the Auxiliary Activities class of the CAZy database (Lombard et al., 2014), are flavoproteins typically constructed of FAD-binding domain and the substrate-binding domain. Most of the fungal GMC oxidoreductases are secreted, extracellular enzymes and oxidize chemically diverse electron donor substrates (Martínez et al., 2009; Lundell et al., 2014; Ferreira et al., 2015; Sützl et al., 2018; Karppi et al., 2020). They are divided into oxidases and dehydrogenases based on the ability (oxidases) or inability/inefficiency (dehydrogenases) to use oxygen as the final electron acceptor (Sützl et al., 2018, 2019).

As a result of GMC enzyme activity, extracellular H_2_O_2_ is produced, which can furthermore promote peroxidase activity and formation of radicals in fungal degradative processes (Figure 1). The AA3 CAZy family is divided into four subfamilies: AA3_1 including cellobiose dehydrogenase (CDH, EC 1.1.99.18); AA3_2 including aryl-alcohol oxidase (AAO, EC 1.1.3.7), glucose oxidase (GOx, EC 1.1.3.4), glucose dehydrogenase (GDH, EC 1.1.5.9) and pyranose dehydrogenase (PDH, EC 1.1.99.29); AA3_3 including alcohol (methanol) oxidase (AOx, EC 1.1.3.13); AA3_4 including pyranose oxidase (POx, EC 1.1.3.10) (Lombard et al., 2014; Sützl et al., 2019) (Table 1).

According to our bioinformatic search, all GMC-AA3 oxidoreductase-encoding genes are much less prevalent in the *Basidiomycota* classes *Dacrymycetes, Tremellomycetes*, and *Wallemiomycetes* as well as in the subphyla *Ustilaginomycotina* and *Pucciniomycotina*, compared to species of *Agaricomycetes* (Figure 2A, Supplementary Table 1). Expansion of GMC-AA3 genes in *Agaricomycetes* was recently discussed in an extensive comparative genomics study with suggestion for their role in fungal development (Krizsán et al., 2019).

The AA3_2 enzyme aryl-alcohol oxidase (AAO) is a secreted enzyme acting to produce extracellular H_2_O_2_ or acting as potential reductant in redox-coupled electron transfer for other oxidoreductases (Martínez et al., 2009; Ferreira et al., 2015; Bissaro et al., 2018). Based on the localization predictions, this holds true for a majority (83%) of the protein models classified as aryl-alcohol oxidase in this study. For the predicted homologs of pyranose dehydrogenase (PDH), however, only 17 genes remained among the *Basidiomycota* genomes after identity threshold filtering. In agreement with a recent study (Sützl et al., 2019), putative PDH were found in species of *Agaricomycetes* family *Agaricaceae* (*Agaricus bisporus* and *Coprinopsis cinerea* in this case), and the predicted proteins all demonstrated extracellular localization (secretion signal with one exception) (Figure 2A, Supplementary Table 1).

Among the alcohol oxidase (AOx) candidates remaining after identity threshold filtering, a majority (95%, 188 genes) were putatively peroxisomal (Supplementary Table 1). Most of the *Basidiomycota* in this study possess several AOx homologs (Figure 2A)—one with high identity to the *Phanerochaete chrysosporium* AOx query sequence (52–91% amino-acid sequence identity) together with one or more homologs with lower identity (35–46%) (Supplementary Table 1). Multiple sequence alignment confirmed that the high-identity proteins correspond to AOx whereas the low-identity candidates are AOx-like proteins (Sützl et al., 2019). Only a handful of species have more than one AOx, while the presence of several AOx-like proteins is more common. However, except for *Boletus edulis*, all studied ectomycorrhizal symbiotic species of *Agaricales, Boletales*, and *Russulales* are deficit of AOx encoding genes, but instead possess 2–9 AOx-like protein encoding genes (Figure 2A, Supplementary Table 1).

The AA3_1 enzyme cellobiose dehydrogenase (CDH) is considered a natural redox partner of the CAZy family AA9 lytic polysaccharide monooxygenases (reviewed in Bissaro et al., 2018). CDH is an extracellular multidomain enzyme composed of a flavin-binding GMC dehydrogenase fused to a heme-binding cytochrome (Cyt) domain, and in some instances, to a carbohydrate-binding module (CBM) (Sützl et al., 2018, 2019). Only a moderate abundance of genes for putative CDH homologs were found among the studied *Basidiomycota* (Figure 2A, Supplementary Table 1), and most of them were predictably extracellular. A majority had both cytochrome and dehydrogenase domains, while some contained the flavin-binding dehydrogenase domain only. It has been suggested that the fungal CDH enzymes might function without a Cyt domain (Sützl et al., 2019).

In our study, *Tulasnella calospora* of *Cantharellales* was the only symbiotic mycorrhizal species that possesses genes for putative CDH enzymes (3 genes), similar to the saprobic *Botryobasidium botryosum* of the same order (Figure 2A, Supplementary Table 1). The brown rot fungi of *Polyporales* lack genes for CDH homologs, on the contrary to the brown rot species of *Agaricales* and *Boletales*. This is in line with previous studies (Floudas et al., 2012; Ferreira et al., 2015). Ignoring the taxonomically restricted PDH (Sützl et al., 2019), pyranose oxidase POx was the most poorly represented GMC oxidoreductase in the set of *Basidiomycota* genomes with only 25 genes predicted as scattered within the class *Agaricomycetes* (Figure 2A, Supplementary Table 1). Among the candidate POx, two thirds may have peroxisomal localization, while the remaining protein models show primarily mitochondrial localization by prediction.

##### Copper-Radical Oxidases

Glyoxal oxidase (GLOX) belongs to the enzyme family of copper radical oxidases (CROs) including enzymes like galactose oxidase (GAOX, EC 1.1.3.9) (Yin D. et al., 2015) (Table 1). Glyoxal oxidases are also classified into the CAZy database auxiliary enzymes in family AA5_1 (Lombard et al., 2014). GLOX reduces O_2_ to H_2_O_2_, and it has broad substrate specificity for the oxidation of simple aldehydes, such as glyoxal and methylglyoxal, to the corresponding carboxylic acids (Kersten and Cullen, 2014).

GLOX and GAOX are copper metalloenzymes containing an unusual free radical-coupled Cu atom in the active site (Kersten and Cullen, 2014; Daou and Faulds, 2017). Five CRO protein subfamilies have been recognized in the *Basidiomycota* class *Agaricomycetes* (Kersten and Cullen, 2014). These subfamilies are based on the putative CRO proteins identified in *P. chrysosporium*: CRO1, CRO2, CRO3–5, CRO6, and GLOX (Vanden Wymelenberg et al., 2006). The proteins in the CRO3–5 subfamily have N-terminal tandem copies of the WSC (cell-wall integrity and stress-response component) domain, suggested to be involved in carbohydrate binding (Vanden Wymelenberg et al., 2006).

In our bioinformatics analysis, genes coding for GLOX and CRO3–5 subfamily proteins were identified among *Agaricomycetes* species only (Figure 2A, Supplementary Table 1). One or several GLOX-encoding genes were identified in more than half of the white rot species, while none was found among the brown rot species. The presence/absence and number of genes coding for CRO1, CRO2 and CRO6 seemed more dependent on taxonomic grouping than on fungal lifestyle (see also **Figure 4B**).

#### Extracellular ROS Utilization

##### Oxidoreductases in Degradation of Plant Biomass

###### Laccases.

Laccases are phenol-oxidizing (EC 1.10.3.2) oxidoreductases belonging to the protein superfamily of multicopper oxidases (MCOs) (Hoegger et al., 2006; Hildén et al., 2009), and are classified into family AA1 in the CAZy database (Lombard et al., 2014). Laccases reduce molecular oxygen O_2_ into two water molecules by four electrons requiring a series of electron transfer from the reducing (electron donating) substrate molecules, which generally are organic and phenolic compounds (Hildén et al., 2009; Lundell et al., 2010) (Table 1). In these reactions, usually phenoxy radicals are formed. Laccases may extend their oxidative potential by oxidation of low molecular weight aromatic mediator compounds into radicals, which subsequently may oxidize macromolecular compounds like polyphenols, lignin, humic substances, and polymeric dyes (Agustin et al., 2021).

By oxidation of phenolic compounds, fungal secreted laccases may have a role in supporting non-enzymatic radical reactions and ROS-generating Fenton chemistry (Jensen et al., 2001; Arantes and Goodell, 2014) (Table 1). In this sense, laccase may be described as an extracellular ROS quenching enzyme, as well as a radical-producing and ROS-generation supporting enzyme (Figure 1). Due to its indirect role in fungal ROS chemistry—not being directly involved in either generation or quenching of reactive oxygen species—laccase encoding genes were not included in our bioinformatic analysis.

###### Class-II Peroxidases.

Lignin-oxidizing and modifying class-II peroxidases of fungi are the enzymes lignin peroxidase (LiP, EC 1.11.1.14), manganese peroxidase (MnP, EC 1.11.1.13) and versatile peroxidase (VP, EC 1.11.1.16) (Table 1), which belong to the superfamily of heme-peroxidases (current synonym is peroxidase-catalase superfamily) and its subfamily class-II of fungal secreted peroxidases (Welinder, 1992; Hofrichter et al., 2010; Zamocky et al., 2014), and are classified into the family AA2 of the CAZy database (Lombard et al., 2014). In addition, a recent phylogenetic analysis suggests six new sub-classes of class-II peroxidases among fungi of *Ascomycota* and *Basidiomycota* (Mathé et al., 2019).

The high-redox class-II peroxidases (AA2 PODs) and their respective genes are found in the white rot and litter-decomposing fungi, and in a few ectomycorrhizal (ECM) species of the *Basidiomycota* class *Agaricomycetes* (Floudas et al., 2012; Lundell et al., 2014; Riley et al., 2014; Kohler et al., 2015). Class-II peroxidases are secreted and glycosylated, globular proteins with a heme (protoporphyrin-IX) cofactor as the electron transfer center (Ruiz-Dueñas and Martínez, 2009; Hofrichter et al., 2010). MnP and VP enzymes contain a Mn binding site, whereas LiP and VP have a specific tryptophan radical center on the surface of the protein (Ruiz-Dueñas and Martínez, 2009; Hofrichter et al., 2010; Lundell et al., 2010; Ruiz-Dueñas et al., 2013). MnP enzymes predicted in the genomes vary substantially, and they can be short, long, extra-long or atypical enzymes (Ruiz-Dueñas et al., 2013).

Hydrogen peroxide is used as an electron acceptor during catalysis of class-II PODs, with a release of two water molecules and two-electron oxidation of the second, reducing substrate, which is an aromatic compound for LiP, whereas MnP and VP may become reduced by Mn^2+^ ions (Hofrichter et al., 2010) (Table 1). Class-II peroxidases require acidic conditions to function. By production of aromatic radicals (in LiP and VP catalysis) and Mn^3+^ chelates (in MnP and VP catalysis), PODs extend ROS-activated oxidative reactions into plant biomass lignocellulose, mainly oxidizing and fragmenting the lignin oligomers (Hofrichter et al., 2010; Kersten and Cullen, 2014; Lundell et al., 2014) (Figure 1). In laboratory cultivations, excess atmospheric dioxygen and ROS induce specific expression of a LiP encoding gene (LiP-H2) in *P. chrysosporium* (Belinky et al., 2003).

In our bioinformatic analysis, AA2 PODs were found in multigene families only within the *Basidiomycota* class *Agaricomycetes*, but not among all orders or fungal species (Figure 2A, Supplementary Table 1). Highest number of POD encoding genes (even over 20 genes) was found among the white rot species of *Polyporales, Hymenochaetales* and *Auriculariales*, and in the litter-decomposing species of *Agaricales*. Noticeably, brown rot fungi of various taxonomic orders lack typical high-redox potential PODs, but instead, may involve 1-2 gene models for homologs of low-redox potential, generic class-II heme-peroxidases (Floudas et al., 2012; Ruiz-Dueñas et al., 2013; Riley et al., 2014). Ectomycorrhizal symbiotic species of the class *Agaricales* generally lack PODs (Floudas et al., 2012; Kohler et al., 2015; Miyauchi et al., 2020) except in a few species, as in our case was detected in *Cortinarius glaucopus* possessing 12 candidate genes for class-II peroxidases (Figure 2A, Supplementary Table 1).

###### Dye-Decolorizing and Heme-Thiolate Peroxidases.

In addition to class-II peroxidases, dye-decolorizing peroxidases (DyP, EC 1.11.1.19) and heme-thiolate peroxidases (HTP, EC 1.11.2.1) (Table 1) are found among the saprobic *Agaricomycetes* (Figure 2A). These heme-including peroxidases are secreted enzymes and belong to different groups within the heme-peroxidase superfamily.

DyPs are ubiquitous enzymes which are found, in addition to fungi, in plants, insects and bacteria (Hofrichter et al., 2010; Linde et al., 2015; Sugano and Yoshida, 2021). As enzymes, they reduce hydrogen peroxide to water and oxidize phenolic compounds as well as LiP-enzyme substrates like non-phenolic lignin model dimers and veratryl alcohol, but in much slower catalytic efficiency and reactivity (Salvachúa et al., 2013; Linde et al., 2015; Lundell et al., 2017). In our bioinformatic analysis, a few DyP encoding genes were found per fungal genome among *Agaricomycetes* and *Pucciniomycotina* (Figure 2A, Supplementary Table 1).

HTPs are hybrid peroxidase-peroxygenase oxidoreductases including unspecific peroxygenases (APO/UPO) and chloroperoxidase (CPO)-like enzymes, which utilize hydrogen peroxide as an oxidant and oxygen donor (Hofrichter et al., 2010) (Table 1). HTPs may act as oxygenating and hydroxylating enzymes, but they also oxidize veratryl alcohol and aromatic compounds which may originate from lignin or plant extractives (Ullrich et al., 2004; Pecyna et al., 2009).

In our bioinformatic analysis, APO/UPO protein encoding genes were identified only in the class *Agaricomycetes* in symbiotic mycorrhizal and litter-decomposing species of the orders *Agaricales* and *Boletales* as well as in the wood-inhabiting species *Auricularia subglabra* (Figure 2A, Supplementary Table 1). Putative HTPs were divided into APO/UPO and CPO-like proteins (Pecyna et al., 2009; Faiza et al., 2019). Genes for putative CPO candidates, on the other hand, were evenly distributed among the taxonomic orders of *Basidiomycota* (Figure 2). Some *Basidiomycota* species possessed genes putatively encoding both types of HTP enzymes, which has been reported previously for *Agaricus bisporus* (Morin et al., 2012). Some species had either APO/UPO or CPO encoding genes while in the third group of fungi, no genes for heme-thiolate enzyme proteins could be identified at all (Figure 2A, Supplementary Table 1).

###### Lytic Polysaccharide Monooxygenases.

Lytic polysaccharide monooxygenases (LPMOs, EC 1.14.99.54, EC 1.14.99.56) of the CAZy family AA9 and a few other AA families oxidize and cleave glycosidic bonds in polysaccharides (Lombard et al., 2014) (Table 1). LPMO enzymes contain one Cu atom, and are found in fungi, bacteria, and animals (Tandrup et al., 2018). LPMO enzymes may become oxidized either by O_2_ or H_2_O_2_ and act as monooxygenases or peroxygenases, respectively, delivering one O atom into the reducing carbohydrate oligomeric substrate, thereby cleaving the glycosidic bond (Bissaro et al., 2018; Tandrup et al., 2018) (Figure 1).

For LPMO catalysis by oxygen activation, formation of intermediate H_2_O_2_ has been demonstrated (Wang et al., 2019), and these enzymes may also become directly activated by light (Bissaro et al., 2020). It has been proposed that LPMO enzymes may promote catalytic activity of AA2 class-II peroxidases and oxidation of lignin-like compounds (Li et al., 2019). Other studies have shown that LPMOs can use lignin derivatives as reductants in reactions of decomposition of cellulose (Kracher et al., 2016; Brenelli et al., 2018).

In our bioinformatic analysis, most of the candidate proteins (97%) for LPMOs showed predicted extracellular localization (Figure 2A, Supplementary Table 1). In all, our searches recognized putative LPMO encoding genes in *Basidiomycota* more in line with the results of Couturier et al. (2018) than was found in an earlier bioinformatic survey (Busk and Lange, 2015).

The number of LPMO encoding genes present in each genome apparently depends both on taxonomic grouping and lifestyle. For example, with the exception of the orchid mycorrhizal species *Tulasnella calospora*, the symbiotic fungi possess very few genes coding for LPMOs (Figure 2A, Supplementary Table 1). Among *Agaricomycetes*, the brown rot species have only a few LPMO encoding genes, except for *Coniophora puteana*, while the white rot species of the order *Polyporales* and the litter-decomposing fungi of *Agaricales* possess high numbers (8–30) of candidate genes for LPMO enzymes. *Agaricomycetes* soft rot of wood—intermediate decay type species have many genes for putative LPMOs, while most of the plant or human-pathogenic species genomes are devoid of these genes. In this respect, abundance of LPMO encoding genes correlates with wood-decaying white rot and litter-decomposing, plant-biomass degrading independent saprobic lifestyles in fungi of *Basidiomycota* (chapter 4.1, **Figure 4B**).

##### Non-enzymatic Fenton Chemistry and ROS Reactions

In Fenton chemistry, soluble Fe^2+^ ions and H_2_O_2_ are needed for generation of ROS, in this case the free hydroxyl radicals (^−•^OH) (Table 1). It is implicated that hydroxyl radicals are the major oxidants to contribute to depolymerization of polysaccharides (cellulose and hemicelluloses) in brown rot decay of wood (Kerem et al., 1999; Jensen et al., 2001; Arantes and Goodell, 2014). Iron Fe^2+^ is sequestered from the solid substrates by reduction and chelation with fungal secreted oxalate or siderophore secondary metabolites (Arantes and Goodell, 2014; Lundell et al., 2014; Keller, 2019) (Figure 1).

For functional Fenton chemistry reactions (H_2_O_2_ + Fe^2+^ + H^+^ -> H_2_O + Fe^3+^ + OH), acidic conditions are a prerequisite (Prousek, 2007). Furthermore, the generated Fe^3+^ ions must be reduced back to Fe^2+^ together with a constant supply of H_2_O_2_. Hydrogen peroxide may be supplied enzymatically by the various AA3 GMC oxidases and AA5 copper-radical oxidases like GLOX, coupled NOX-SOD activity, through translocation, or non-enzymatically (Figure 1). Thus, operation of effective Fenton chemistry may involve participation of fungal ROS producing oxidoreductases. For effective brown rot decay of wood by Fenton chemistry several mechanisms have been proposed (Kerem et al., 1999; Jensen et al., 2001; Hammel et al., 2002; Arantes and Goodell, 2014; Zhang et al., 2016; Shah et al., 2018). For supply of H_2_O_2_, specific AA3-GMC AOx methanol-oxidases have been suggested to operate in the brown rot species *Gloeophyllum trabeum* (Daniel et al., 2007), *Serpula lacrymans* (Eastwood et al., 2011) and *Rhodonia (Postia) placenta* (Vanden Wymelenberg et al., 2011) of *Basidiomycota*.

Laboratory experiments have suggested that fungal created Fenton chemistry could operate by the assistance of low molecular weight organic compounds, especially phenols and quinones, which may be produced by the fungi or generated from decomposing plant biomass polyphenols, wood lignin, and other aromatic units (Kerem et al., 1999; Hammel et al., 2002; Arantes and Goodell, 2014). In these phenol-quinone recycling reactions, secreted laccase enzymes may be involved (Jensen et al., 2001).

Brown rot fungi also secrete polysaccharide-degrading CAZymes, which in turn may be sensitive to ROS and radicals created by the Fenton reactions (Kerem et al., 1999; Martínez et al., 2009; Ryu et al., 2011). In the so called “staggered mechanism” of brown rot, at first an oxidative attack leads to loosening of the wood cell wall lignocellulose structure. Temporal (time-based) separation of the two mechanisms (radical-driven oxidation phase and later enzymatic degradation) has been demonstrated for the brown rot fungi *R. placenta* and *G. trabeum* (Zhang et al., 2016; Presley et al., 2018).

## Part II: Intracellular ROS

### Intracellular ROS in *Basidiomycota*

**Figure 3:**
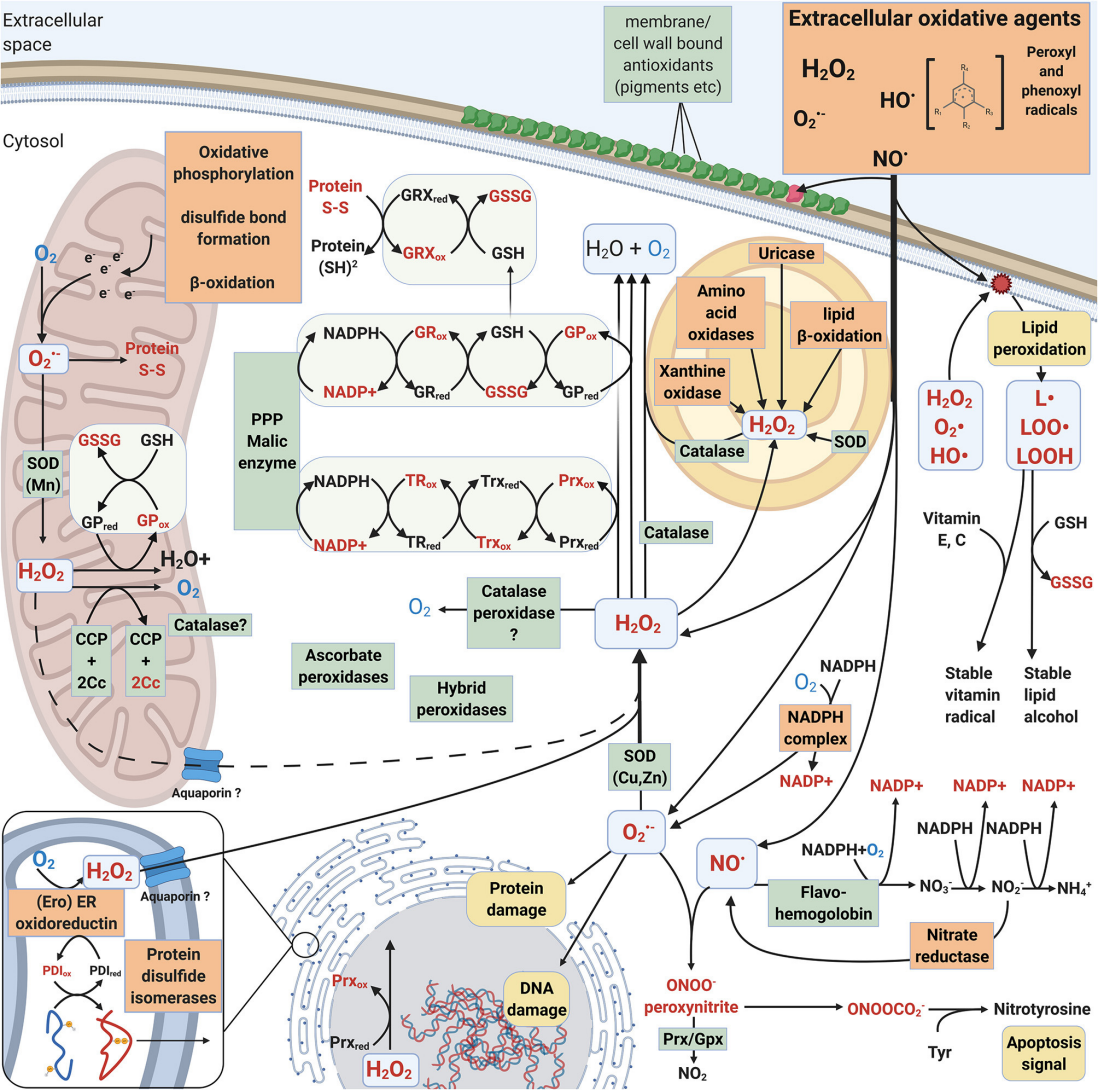
Intracellular mechanisms involved in generation and mitigation of oxidative stress created by ROS and RNS. Oxidized compounds, radicals and proteins are indicated in red. L• = Lipid radical, LOO• = Lipid peroxyl radical, LOOH = lipid hydroperoxide. Mechanisms that increase oxidative stress are boxed and highlighted with orange. Mechanisms that decrease oxidative stress are pointed out in green boxes.

The main difference between intracellular and extracellular ROS actions in fungi is that many extracellular reactions are activated by ROS whereas intracellular responses and pathways are either activated by ROS or create ROS as side products. Essential intracellular activities such as protein maturation in the endoplasmic reticulum (ER), respiratory electron transfer pathway in the mitochondrial inner membranes, and oxidation reactions in the peroxisomes create ROS (Schrader and Fahimi, 2006; Jastroch et al., 2010; Sibirny, 2016) (Figure 3).

#### Sources of Intracellular ROS in Fungi

Typically, superoxide and hydrogen peroxide are formed during cellular metabolism and energy conversion. Respiratory electron transfer chain in mitochondria is the principal generator for intracellular ROS and oxidative stress in eukaryotes. During oxidative phosphorylation electrons are leaked for instance from complex III (cytochrome *c*reductase) leading to formation of superoxide radicals when transferred to the terminal oxygen molecule at complex IV (Jastroch et al., 2010). In plants, corresponding electron leakage in chloroplasts—especially under excess light conditions—results in ROS formation and requires efficient antioxidant enzymes analogous to mitochondrial defense.

In cellular metabolism, β-oxidation is responsible of degradation of fatty acids to acetyl-CoA to produce energy (Schrader and Fahimi, 2006; Sibirny, 2016). In peroxisomes, β-oxidation forms hydrogen peroxide whereas in mitochondria, the excess of electrons can lead to formation of superoxide when reacting with molecular oxygen (Figure 3). In the *Ascomycota* yeast *Saccharomyces cerevisiae*, β-oxidation performing acyl-CoA oxidase enzymes function in the peroxisomes (Sibirny, 2016). In a few *Basidiomycota* such as the *Agaricomycetes* mycorrhizal species *Laccaria bicolor* and the plant pathogen *Ustilago maydis* of *Ustilaginomycotina*, both mitochondrial and peroxisomal β-oxidation mechanisms have been recognized (Reich et al., 2009; Kretschmer et al., 2012).

Protein folding and post-translational maturation in the ER requires an oxidative agent such as molecular oxygen to introduce disulphide bonds. This process leads into the formation of hydrogen peroxide (Figure 3). Aquaporin (Aqp11) has been proposed to be responsible for translocation of H_2_O_2_ between the ER and cytosol (Rashdan and Pattillo, 2020). Together with mitochondria, ER is thereby one of the central cellular organelles generating ROS (Laurindo et al., 2014). Disruption of cellular iron sulfur clusters (present and functional in protein folding in the ER, mitochondrial electron transfer chain, and in antioxidant enzymes) release Fe^2+^ ions which can react with O_2_ or H_2_O_2_ and form ROS—superoxide or hydroxyl radicals, respectively. This would lead to destructive consequences if allowed to occur intracellularly.

Xanthine oxidase/dehydrogenase/oxidoreductase (XO, XOR, EC 1.17.3.2) is a ROS-producing intracellular iron-molybdenum flavoprotein enzyme containing iron-sulfur clusters (Harrison, 2002). XO is functional in recycling of purine derivatives by degradation of xanthine or hypoxanthine to uric acid (Figure 3).

Like XO, the next enzyme uricase utilizes molecular oxygen and produces H_2_O_2_ as a side product (Figure 3). The animal-pathogenic *C. neoformans*, which is commonly isolated from uric acid rich pigeon guano, may catabolise uric acid into ammonia (Lee et al., 2013). Unicellular yeasts such as *S. cerevisiae* and *Ascomycota* filamentous fungi in turn are rich in uric acid (Hafez et al., 2017). These findings, together with our genomic analyses, imply differences in purine catabolism and intracellular ROS generation between *Ascomycota* and *Basidiomycota*.

#### Intracellular ROS Mitigating Enzymes

##### Superoxide Dismutase

Superoxide ${\text{O}}_{2}^{-\cdot }$ is abundant in the environment of aerobic organisms as well as generated intracellularly as a by-product in molecular oxygen-involving reactions. Therefore, organisms that reside in aerobic environments usually apply superoxide dismutase (SOD) activity to mitigate oxidative damage. The source of ${\text{O}}_{2}^{-\cdot }$ may vary from endogenous metabolism to exogenous pathogenic defense reactions, often produced by NOX (chapter 2.1.1, Table 1). Intracellular ${\text{O}}_{2}^{-\cdot }$ is needed in low concentrations for constant production of H_2_O_2_, e.g., for protein folding in the ER as well as for signaling (D'Autreaux and Toledano, 2007; Halliwell and Gutteridge, 2015; Rashdan and Pattillo, 2020). SOD, together with catalase (CAT) are considered the first line of antioxidant defense toward exogenous ROS inside the cells (Packer and Cadenas, 2007; Schatzman and Culotta, 2018) (Figure 3). However, ROS defense mechanisms have redundancy, and for instance SOD and CAT functions may be replaced by other enzymes and oxidative stress quenching proteins (see below).

Superoxide dismutases (SOD) (EC 1.15.1.1) are antioxidant metalloproteins in which the coordinated Fe, Mn, Cu-Zn, or Ni atoms are forming the redox center in the active enzyme (Wuerges et al., 2004; Schatzman and Culotta, 2018). According to protein structure and metal cofactor, three SOD families are recognized. In eukaryotes, SOD is located in the cytosol as well as inside mitochondria and other organelles like peroxisomes and chloroplasts (Schatzman and Culotta, 2018). In bacteria, extracellular SODs may be located into the periplasmic space. Function of the SOD enzyme is to dismutate (and quench) superoxide ${\text{O}}_{2}^{-\cdot }$ radicals by reduction to O_2_ and H_2_O_2_ (McCord and Fridovich, 1969).

In fungi, SOD1 usually refers to the cytosolic and mitochondrial intermembrane-space located Cu/Zn-SOD, whereas SOD2 refers to a mitochondrial matrix located Mn-SOD enzyme (Schatzman and Culotta, 2018). The SOD3 variant represents a cytosolic Mn-SOD found especially in the opportunistic humanpathogen *Ascomycota* species *Candida albicans* (Li et al., 2015) whereas the SOD4–6 are secreted monomeric Cu-SOD enzymes. However, many of them include an GPI (glycosylphosphatidylinositol) anchor, thereby becoming attached to the fungal cell wall (Youseff et al., 2012; Schatzman and Culotta, 2018). It should be noted, however, that the naming of SODs is not uniform, and so the type of SOD cannot always be deduced from the enzyme or gene abbreviation or name. For instance, in the plant-pathogenic species *Puccinia striiformis* of *Basidiomycota*, there is a *PsSOD1* gene that encodes a secreted Zn-only SOD (Liu et al., 2016).

Based on our genomic analysis the number of putative SOD homolog encoding genes per fungal genome varied from two up to 15 candidates (Figure 2B, Supplementary Table 1). In most genomes, the number of SOD encoding genes ranged between 2 and 6. However, no clear correlation was found between the number of putative SOD candidates and different lifestyles among the species of *Basidiomycota*. Based on *in silico* prediction, SOD1 proteins (Cu/Zn-SODs) are mainly located into vacuoles or in the cytoplasm, but some may become secreted and extracellular. Cytoplasmic localization of SOD1 was predicted for species of *Dacrymycetes* and *Tremellomycetes*. Fungi in *Wallemiomycetes, Ustilaginomycotina* and *Puccioniomycotina* lacked SOD1 homologs, except in *Septobasidium* sp. PNB30-8B.

In the animal-pathogenic fungus *C. neoformans*, SOD1 is described as a cytosolic enzyme, although SOD activity is also detected in the lipid rafts of its plasma membrane (Siafakas et al., 2006), and vacuolar localization may occur under oxidative stress conditions (Kim et al., 2021). In *C. neoformans*, the Cu-sensing transcription factor Cuf1 regulates expression of SOD encoding genes, and a novel cytosolic isoform of SOD2 under Cu-limitation (Smith et al., 2021). In *Puccinia striiformis* f. sp. *tritici*, the extracellularly functioning Zn-only SOD has an important role in early infection of the wheat plant host (Liu et al., 2016). Moreover, *P. striiformis f. sp. tritici* possesses an additional Cu-only SOD (Zheng et al., 2020).

Homologs of SOD2 and SOD3 were detected in all investigated species of *Basidiomycota*, commonly one to four genes per genome encoding these enzyme variants (Figure 2B, Supplementary Table 1). Thereby it may be concluded that fungi of *Basidiomycota* apparently possess at least one mitochondrial SOD2/3. When several genes encoding SOD2/3 were found, they were typically proteins with both mitochondrial and cytoplasmic localizations predicted. On the contrary, putative SOD4/5/6 proteins were detected only in two species of *Agaricomycetes* in the order *Cantharellales* (*Botryobasidium botryosum* and *Tulasnella calospora*), in *Dacrymycetes* (*Dacrymyces fennicus*) and in *Pucciniomycotina*. Localization of these proteins was predicted either to the extracellular space or as integrated into the cell membrane.

##### SOD Expression in Pathogenic Basidiomycota

Plant pathogenic fungi experience oxidative stress during infection of the host (Heller and Tudzynski, 2011). In the *Agaricomycetes* conifer-tree pathogen species *Heterobasidion annosum*, expression of the gene encoding SOD1 was increasing during early infection of Scots pine (Karlsson et al., 2005). The Norway spruce-specific necrotrophic pathogen *Heterobasidion parviporum* showed similar induction of gene expression immediately after inoculation. Shifts in the *SOD* gene transcript levels may follow the nutritional status of the fungus during its mycelial growth inside the host woody tissue (Karlsson et al., 2005).

In the animal pathogen *C. neoformans*, the Cu/Zn-SOD1 variant is critical for virulence (Warris and Ballou, 2019). *C. neoformans* encounters both higher environmental temperature when entering the animal host as well as additional oxidative stress by engulfment and action of macrophages (by NOX). In microarray studies on *C. neoformans*, a Mn-SOD encoding gene was identified as induced in expression at +37°C (Kraus et al., 2004) and a robust transient transcriptional response was observed under oxidative stress generated by treatment with H_2_O_2_ (Upadhya et al., 2013). In the latter study, downregulation of a Cu/Zn-SOD (SODC) encoding gene was noticed. In contrast, the mitochondrial Mn-SOD2 enzyme is explained to be required for detoxification of ROS generated by augmented respiration at elevated temperatures (37°C) (Giles et al., 2005). SOD has also been detected in extracellular vesicles secreted by *C. neoformans* (Wolf et al., 2014). In conclusion, each *C. neoformans* SOD enzyme type and respective gene seemingly respond specifically to stress by heat and oxidative agents.

There are differences between animal-pathogenic fungi of *Ascomycota* and *Basidiomycota* when it comes to the functions of the various SOD homologs (Warris and Ballou, 2019). Animal-pathogenic fungi also need to survive from the macrophage oxidative burst (of superoxide, hydrogen peroxide and NO) upon infection and invading the host. High SOD activity prevents the formation of peroxynitrites between NO and ${\text{O}}_{2}^{-\cdot }$. In *C. neoformans*, SOD-generated hydrogen peroxide is quenched by the glutathione system and glutathione peroxidase (GPX) instead of catalases (Missall et al., 2005), which is different from the SOD-CAT system functional in the opportunistic species *C. albicans* and *Aspergillus fumigatus* of *Ascomycota*. Thereby, assumingly redundant antioxidant mechanisms have evolved and adapted in the animal-pathogenic fungi of *Ascomycota* and *Basidiomycota*.

##### NADPH-Dependent Thiol-Mediated Antioxidant Systems

Glutaredoxin (Grx) and glutathione peroxidase (GPx) together with glutathione reductase (GR) form the NADPH-dependent glutathione system. Thioredoxin (Trx), peroxiredoxin (Prx), and thioredoxin reductase (TR or TrxR) form the thioredoxin system. These thiol-dependent redox enzymes and assisting small proteins are responsible for quenching of intracellular ROS and build up the effective cellular defense system in living organisms against oxidative stress (Morel et al., 2008; Balsera and Buchanan, 2019; Ulrich and Jakob, 2019). In this review, we especially focus on the presence of genes encoding these essential antioxidant proteins in species of *Basidiomycota* representing different lifestyles and nutritional traits.

###### Thioredoxin and Peroxiredoxin.

Thioredoxin (Trx) is a small oxidoreductase enzyme containing an active site for dithiol-disulphide exchange, with the main function to reduce the oxidized form of peroxiredoxin (Prx) (Figure 3). The Trx active site contains a redox-active disulphide bond surrounded by well-conserved amino acids, generally forming the motif WCGPC (Balsera and Buchanan, 2019). In our analysis, the most common active site motif among the studied *Basidiomycota* was the conserved WCGPC. We observed three different types of Trx proteins: a mitochondrial Trx and two very similar Trx protein types which differed in their C-terminal ends. We assigned these Trx types the working names “Trx short” and “Trx long,” reflecting the fact that one was longer than the other (Figure 2B). However, we did not observe any correlation between taxonomic grouping or fungal lifestyle and the copy number or type of Trx-encoding genes.

Oxidized Trx is reduced back by thioredoxin reductase (TR) (Figure 3). Putative TR encoding genes were found in all studied *Basidiomycota* genomes (Figure 2B, Supplementary Table 1). Most of the *Basidiomycota* species possess a single gene for putative TR enzyme. The TR homologs of *Pycnoporus cinnabarinus* and *Russula brevipes* both lacked the NADPH-binding domain but were included in the analysis since the FAD-binding domain and enzyme active site were present (Supplementary Table 1). As redox enzymes, TR are structurally and functionally similar to glutathione reductases (GR) and catalyze NADPH-dependent reduction of Trx (Mustacich and Powis, 2000).

###### Peroxiredoxin.

The thioredoxin-dependent peroxiredoxin (Prx) reduces a wide array of ROS as well as alkyl and lipid hydroperoxides (ROOH), with primary function as a peroxidase and sensor for peroxides (Rhee et al., 2012). Oxidized Prx is reduced back by thioredoxin (Trx) (Figure 3). Prx are universal cysteine-containing antioxidant proteins found in all prokaryotes and eukaryotes, and the active form is a homodimer (Rhee et al., 2012). In fungi, four different types of Prx have been identified: 1-Cys Prx, 2-Cys Prx, PrxII, and PrxQ (Morel et al., 2008). Among the different types of Prx, there are three important amino acid residues: the conserved peroxidatic cysteine, a recycling C-terminal cysteine and a catalytic arginine. Moreover, the identified type II Prx proteins can be divided into two subtypes, PrxII.1 and PrxII.2 (Morel et al., 2008).

In agreement with the previous study on *Basidiomycota* thiol-dependent antioxidant enzymes (Morel et al., 2008), both cytosolic and nuclear localization were predicted for the 2-Cys Prx type proteins in our bioinformatic search (2B, Supplementary Table 1), while the main predicted localization for 1-Cys Prx type was in the cytoplasm. Also genes coding for putative PrxQ isoforms, PrxQ1 and PrxQ2, were depicted within the *Basidiomycota* genomes. Both PrxQ types encoding genes were distributed among the different taxonomic groups, with a few exceptions. No PrxQ-encoding genes could be identified within the species of *Wallemiomycetes*, and species of *Pucciniomycotina* possess only PrxQ2 type encoding genes. Localization predictions for these proteins were in agreement with the previous study (Morel et al., 2008); PrxQ1 is predictably functional in the cytoplasm whereas for PrxQ2, a majority of the putative proteins will become nuclear, with a few exceptions (showing cytosolic localization) (Supplementary Table 1). As with other translated protein models in our bioinformatic analyses, however, uncertainties in the N-terminal part of coding sequence for the gene models may have affected the analysis.

Sulfiredoxins (Srx) interact with 2-Cys Prx to reduce sulfinic acids formed on the peroxidatic cysteine (Rhee et al., 2012). It has been suggested that the presence or absence of Srx correlates with the presence of 2-Cys Prx in a few species of *Basidiomycota* (Morel et al., 2008). However, no Srx encoding homologs were identified in species of the class *Agaricomycetes* in our bioinformatic study (Supplementary Table 1).

Methionine sulfoxide reductase (Msr) is involved in the cellular antioxidant system by catalyzing reduction of methionine sulfoxide (MetSO) back to methionine using a thiol-dependent regeneration mechanism (Stadtman et al., 2002). Putative genes for the two types of Msr, MsrA and MsrB, were found in almost all of the studied *Basidiomycota* genomes except for the *Agaricomycetes* species *Trametes versicolor*, missing a MsrA homolog, and *Stereum hirsutum*, missing a MsrB homolog (Supplementary Table 1). However, these exceptions may result from incomplete protein model information.

###### Glutathione System.

Glutathione peroxidase (GPx) functions to eliminate H_2_O_2_ and becomes reduced back to active enzyme by thiol-including molecules such as glutathione (GSH) (Figure 3). There is, however, increasing evidence that fungal GPxs are functionally thioredoxin-dependent peroxidases rather than glutathione-dependent peroxidases (Zhang et al., 2008; Ariadni et al., 2021). An additional conserved cysteine residue, corresponding to Cys88 in the TrGPx of the *Ascomycota* species *Trichoderma reesei*, seems to be involved in the specificity to thioredoxin as a reductant (Ariadni et al., 2021). This additional conserved cysteine was present in all of the predicted GPx proteins of the *Basidiomycota* genomes studied here, thereby indicating specificity more likely toward thioredoxin than glutathione.

In our bioinformatic search, one gene encoding putative GPx was recognized in most of the *Basidiomycota* genomes (Figure 2B, Supplementary Table 1), which is in agreement with previous findings (Morel et al., 2008). However, some correlation between the gene number and fungal taxonomic grouping could be observed: species belonging to the order *Russulales* were devoid of this gene, while species in the class *Tremellomycetes* possess at least two genes for GPx homologs. In the *C. cinerea* Okayama genome there was a candidate gene in which the GPx redox center coding region was missing, thus excluding this protein model in our analysis. In the *C. cinerea* AmutBmut genome, however, a gene for a correct protein model was found (Supplementary Table 1).

In contrast to previous studies where no evidence has been found for other than cytosolic localization for fungal candidate GPx (Missall et al., 2005; Morel et al., 2008), a few of the GPx sequences were predicted for mitochondrial localization in our genome study. In particular, *Tremellomycetes* genomes have one GPx homolog with predicted cytoplasmic localization, together with at least one mitochondrial homolog (Figure 2B, Supplementary Table 1). One explanation is that those fungal genomes with only one gene for GPx could process variants of gene transcripts by alternative splicing, leading to different protein products and cellular localization.

Glutathione reductase (GR) is a flavoprotein responsible for reducing glutathione in an NADPH-dependent manner (Couto et al., 2016) (Figure 3). Function of GR is essential in maintaining the supply of reduced glutathione and cellular redox homeostasis. In our analysis, most *Basidiomycota* genomes had a single gene coding for GR, with a few species possessing 2–4 genes (Figure 2B, Supplementary Table 1). A majority were predicted to become cytosolic (by DeepLoc analysis) but some were potentially plastid-localized. While this is not a sensible prediction in fungi, the same protein sequences were analyzed by another tool, TargetP-2.0 (Supplementary Information File 1, Almagro Armenteros et al., 2019), where most of the proteins with previous “plastid” prediction ended up becoming directed to mitochondria.

In the *Ascomycota* yeast *S. cerevisae* and human cells, a single gene expresses more than one form of GR, which are either directed in the cytoplasm or into various organelles whereas in plants, two genes encoding GR isoforms have been described (Couto et al., 2016). However, in organisms like fungi (and as evidenced by our genome analysis) the parallel thioredoxin-based system may overlap in function with the glutathione antioxidant system (Couto et al., 2016) (Figure 3).

##### Additional Intracellular Peroxidases

Intracellular peroxidases with antioxidant activity include the enzymes cytochrome *c* peroxidase (CCP, CcP, EC 1.11.1.5), ascorbate peroxidase (L-ascorbate peroxidase APX, EC 1.11.1.11) and catalase-peroxidase (EC 1.11.1.21), which all belong to the heme-containing peroxidases of the peroxidase-catalase superfamily (Welinder, 1992; Hofrichter et al., 2010; Zamocky et al., 2014). In fungi, CCP is translocated into the mitochondrial intermembrane space. It is the key enzyme sensing and eliminating respiratory ROS, principally H_2_O_2_, together with the mitochondrial catalases (Martins et al., 2013; Upadhya et al., 2013). In *C. neoformans*, CCP has a protective role against external ROS but is not essential for virulence (Giles et al., 2005).

One gene encoding putative mitochondrial CCP is conserved among *Basidiomycota* according to our genome searches (Figure 2B, Supplementary Table 1). In addition, we detected genes encoding potential cytosolic CCP homologs in fungi of the orders *Tremellomycetes, Wallemiomycetes*, and in *Ustilaginomycotina* and *Pucciniomycotina* (Figure 2B, Supplementary Table 1), which may represent the same findings as reported previously in phylogenetic studies (Zámocký et al., 2012; Zamocky et al., 2014). However, cytosolic catalase-peroxidase enzyme encoding genes were absent in the *Basidiomycota* genomes except in one case, in the plant-pathogenic species *Ustilago maydis*. This enzyme apparently replaces catalase as the primary H_2_O_2_-degrading enzyme in the fungus (Kämper et al., 2006; chapter 3.2.5).

In addition to CCP, the *Basidiomycota* also possess hybrid peroxidases with a predicted extracellular or cell membrane localization (Zamocky et al., 2014). Fungal hybrid peroxidases may represent intermediate enzymes of ascorbate-peroxidase and CCP (type A) or fusions of heme peroxidase to a carbohydrate-binding module (type B) (Zamocky et al., 2014). Type B hybrid peroxidases were found in our analysis within the class *Agaricomycetes* as the most abundant among species of the order *Agaricales* (Figure 2B, Supplementary Table 1).

##### Catalases

Together with SODs, catalases (CAT, EC 1.11.1.6) are the conserved primary antioxidant enzymes keeping the concentrations of intracellular ROS in life-allowing limits. The specific activity of CAT enzymes is reductive disproportionation of excess H_2_O_2_ into water and dioxygen molecules (Alfonso-Prieto et al., 2009). Hydrogen peroxide can enter cells directly through membranes and through aquaporins. CAT are metalloproteins with either Mn or Fe (heme) in their active center and are present in almost all aerobic organisms from prokaryotes to multicellular eukaryotes (Klotz et al., 1997; Zamocky et al., 2008).

CAT active enzymes are usually multimers of similar subunits, and in fungi, several heme-catalase enzymes have been identified. In the filamentous species of *Ascomycota*, heme-catalases of two large-size subunit types (L1 and L2) have been identified together with 1–4 different CAT enzymes of small-subunit types (Hansberg et al., 2012). In the *Ascomycota* yeast *S. cerevisiae*, only small-subunit CAT enzymes are found. The small-size CAT subunits of fungi are related to animal catalase protein subunits. In general, there are three clades of heme-including CAT enzymes, and their evolution is directed from large subunits toward small subunit proteins (Zamocky et al., 2008; Zámocký et al., 2012). Small-subunit CATs can be further divided into NADPH binding and non-binding clades. In addition, the heme orientation is specific to the clades (Hansberg et al., 2012).

It is generally assumed that CAT operate in high H_2_O_2_ concentrations whereas Prx are active at <10 μM concentrations (Díaz et al., 2005; Parsonage et al., 2005). CAT enzymes are located in the cytosol, mitochondria, or peroxisomes (Figure 3), other eukaryotic organelles (like chloroplasts in plants), or may be extracellular like the L2-type catalases of *Ascomycota* fungi (Hansberg et al., 2012).

Previous analyses indicated that many fungi lack peroxisomal CAT (Hansberg et al., 2012). However, in our study we found that most of the analyzed species have a small-subunit CAT with predicted peroxisomal localization (Figure 2B, Supplementary Table 1). Only *Dacryopinax primogenitus, Paxillus involutus*, and *Schizophyllum commune* lacked a peroxisomal SS-CAT. Especially in the mitochondria, CATs act in concert with SODs for quenching of ROS (Figure 3). These two antioxidant enzymes together allow function of the mitochondrial electron transfer chain under aerobic conditions for respiratory generation of cellular energy. Regarding fungal virulence, extracellular CAT enzymes may be involved not only in ROS quenching reactions but act as host-recognized proteins in plant-fungal interactions (Hansberg et al., 2012).

From 1 to 5 genes coding for heme-CAT protein subunits were identified in the *Basidiomycota* genomes (Figure 2B, Supplementary Table 1). One exception was *Ustilago maydis* showing absence of any CAT type encoding genes. Instead*, U. maydis* has one catalase-peroxidase (Kämper et al., 2006). In general, saprobic wood decaying or plant litter-decomposing fungi of *Basidiomycota* possess several genes for both small and large CAT subunit types, but likewise so has the animal-pathogenic species *C. neoformans* (5 genes in *C. neoformans* var. neoformans JEC21; 3 for small and 2 for large subunits) (Supplementary Table 1).

In most cases where only one gene encoding a putative catalase was found in the fungal genome, the predicted protein product was of intracellular small-size subunit SS-CAT type, like in the mycorrhizal species *Laccaria bicolor* and *Fistulina hepatica* (Figure 2B, Supplementary Table 1). On the contrary, the one and only CAT protein in the species of *Tremella* and *Wallemia* is of large-subunit type. Previously, it was noticed that species of *Basidiomycota* have only L1-type catalases, all lacking signal peptide for secretion (Hansberg et al., 2012). The same can be concluded from our analysis, where all of the large-subunit LS-CATs are predictably cytosolic (Supplementary Table 1).

## PART III: Fungal Lifestyles and ROS

### Connection of ROS Enzymes to Lifestyle and Ecology

Expression and activity of superoxide dismutase (SOD) and catalase (CAT) in relation to other fungal oxidoreductase activities has been studied for a few species of *Basidiomycota*. As an example, activities of SOD and CAT were noticed to follow changes in the activities of extracellular lignocellulolytic oxidoreductase enzymes in the *Agaricomycetes* white rot fungi *Phanerochaete chrysosporium* and *Pleurotus sajor-caju* (Pompeu et al., 2019) while cultivated on sugarcane bagasse. However, the authors noted that with increasing extracellular oxidoreductase activities, the intracellular CAT activity decreased. This indicates that production of H_2_O_2_ was permitted to allow supply of this ROS oxidant to the extracellular peroxidases active in depolymerization of the substrate lignocellulose components (Figure 1).

#### Plant-Biomass Degrading Saprobic Fungi and Extracellular ROS

**Figure 4:**
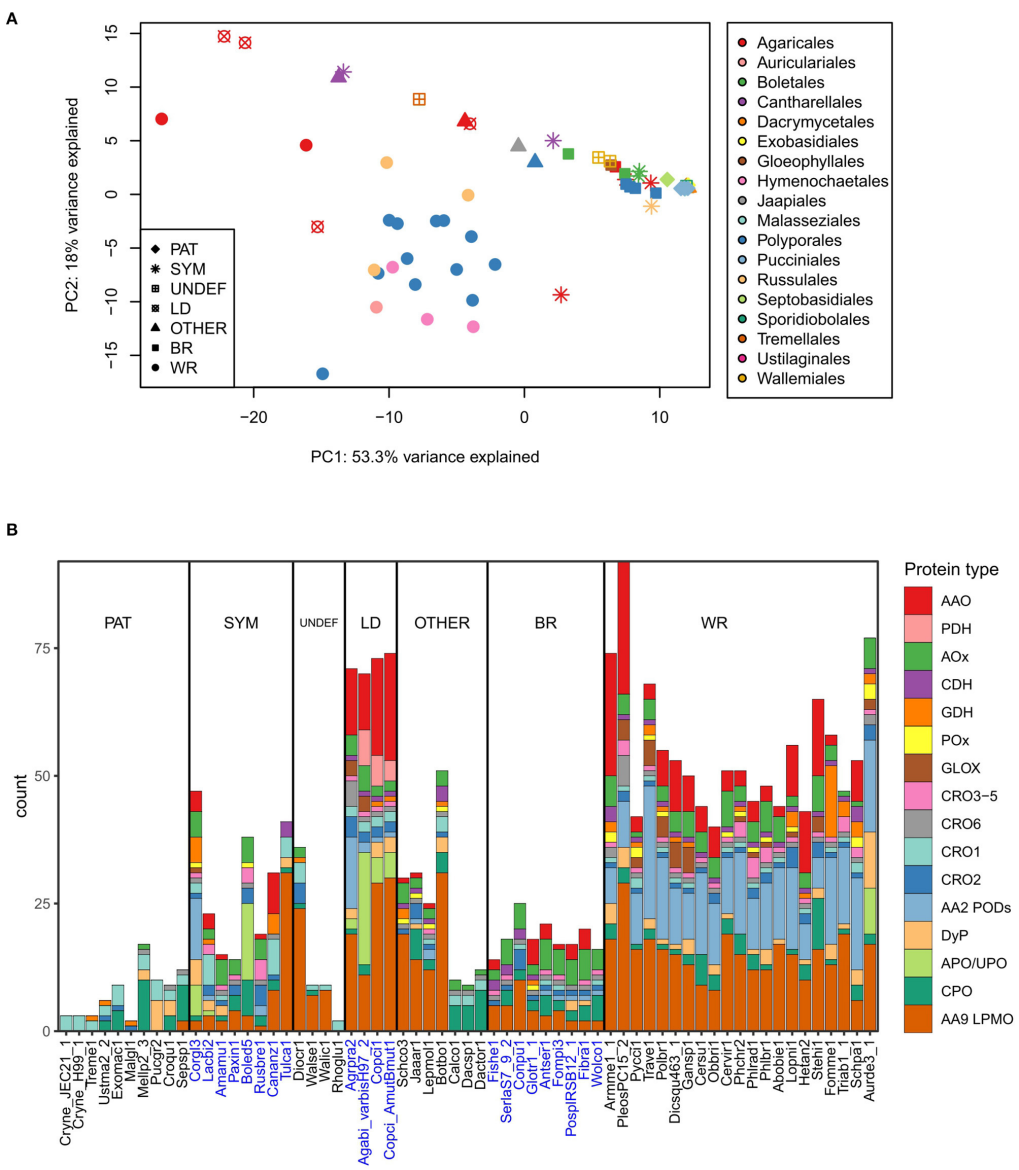
Extracellular ROS generation and utilization-related enzymes in the *Basidiomycota* **(A)** in relation to their lifestyle and order-level taxonomy studied by principal component analysis on gene counts per each genome (top), and **(B)** distribution of genes in each species according to enzyme function and protein family (below). Ecological lifestyle groups: PAT, pathogenic; SYM, symbiotic mycorrhizal; UNDEF, saprobic undefined decomposition type; LD, litter-decomposing; OTHER, other type of wood decay; BR, brown rot; WR, white rot. Protein abbreviations are opened in the text.

In order to visualize potential correlation relationship between fungal lifestyle or taxonomy and enzymes that generate or utilize extracellular ROS, we performed a principal component analysis (Figure 4A) on the gene counts of Figure 2A. The PCA plot shows that wood-decaying white rot species of different taxonomical orders mainly cluster together, whereas most of the brown rot species (also from different taxonomical orders), the symbiotic (mycorrhizal) fungi and the pathogenic species of *Basidiomycota* are found together in a separate cluster (Figure 4A).

This separation can be explained by the high gene count numbers of CAZy AA2 PODs (class-II peroxidases), AA9 LPMOs, and some members of the families of AA3-GMC hydrogen peroxide producing enzymes, in the white rot species (Figure 4B). In this respect, the litter-decomposing species are more similar to white rot fungi than to the other ecological groups. These results are in line with previous comparative genomic studies extensively concentrating on the CAZy enzyme families (Eastwood et al., 2011; Floudas et al., 2012; Riley et al., 2014; Kohler et al., 2015; Nagy et al., 2016; Mäkinen et al., 2019) and confirms the essence of the combination of AA2 class-II peroxidases, several AA3 subfamily enzymes, AA5 CROs and various AA9 LPMOs, all in multigene families, for efficient wood degradation by fungi of white rot decay type.

Lack of AA2 POD genes and reduced number of genes for enzymes of AA9 and AA3 subfamilies in turn explains the similarity of brown rot and symbiotic ectomycorrhizal species (Figure 4B). In all, extracellular ROS enzyme genes were the most substantially reduced in the pathogenic fungi representing all taxonomic classes and subphyla—as well as among *Dacrymycetales* represented by three species in our analysis (Figures 2A, 4B, Supplementary Table 2). Their ROS enzyme gene profile includes chloroperoxidase-like CPO enzymes and a few genes for H_2_O_2_ producing CRO and AA3-AOx. This enzyme pattern is more similar to the extracellular ROS gene set of the plant-pathogenic species *Melampsora larici-populina* of *Pucciniomycotina* than for instance to *Schizophyllum commune* or brown rot fungi of *Agaricomycetes*. In this respect, *Dacrymycetales* may represent another yet undiscovered type of biodegradation of wood and plant litter.

Gene family sizes deviate many of the symbiotic mycorrhizal species, however, away from the brown rot fungal ROS enzyme pattern. Extension in the AA9 LPMO family in the orchid mycorrhizal *Tulasnella calospora* (order *Cantharellales*) is exceptional, as has been noticed in previous studies (Kohler et al., 2015; Miyauchi et al., 2020). Expansion of AA9 family is more similar to the ROS gene patterns illustrated for saprobic lifestyle like *Dioszecia cryoxerica* or other wood decay types like in *Botryobasidium botryosum* (Figures 2A, 4B). The latter species is classified to the same order *Cantharellales* as *T. calospora*. Furthermore, expansion in APO/UPO peroxygenase gene family in *Boletus edulis* is more resembling the litter-decomposing species *Agaricus bisporus*, and in the case of *Cortinarius glaucopus*, similar placing among litter-decomposers could be imagined based on expansion in AA2 POD family (Figures 2A, 4B). These examples confirm that mycorrhizal *Basidiomycota* are not a uniform ecological group. Instead, they owe features of saprobic lifestyle and extracellular ROS gene patterns governed by their taxonomic positioning.

Transcriptomics of wood and plant biomass degrading *Agaricomycetes* species of *Basidiomycota* have revealed concomitant upregulation of genes encoding secreted class-II lignin-modifying peroxidases, lytic polysaccharide monooxygenases, and specific genes encoding H_2_O_2_ producing enzymes of the AA3-GMC superfamily as well as CRO variants (Vanden Wymelenberg et al., 2011; Salvachúa et al., 2013; Hori et al., 2014; Alfaro et al., 2016; Kuuskeri et al., 2016). Extracellular oxidative environment promotes both white rot and brown rot degradative reactions in decomposition of wood lignin and the polysaccharide (cellulose and hemicellulose) polymers (Hammel et al., 2002; Arantes and Goodell, 2014; Lundell et al., 2014; Zhang et al., 2016; Bissaro et al., 2018).

Isolated class-II PODs (LiP, MnP, VP), other extracellular heme-containing peroxidases, and laccases have been extensively studied biochemically and kinetically in their reactivity with H_2_O_2_ and other ROS (reviewed in Martínez et al., 2009; Hofrichter et al., 2010; Lundell et al., 2010; Kersten and Cullen, 2014; Bissaro et al., 2018). Supply of hydrogen peroxide is essential for peroxidase activity, and addition of catalase causes inactivation of POD activity in the reaction mixtures (Tien and Kirk, 1984; Renganathan et al., 1986). Concerted expression of AA3-GMC and AA5-CRO H_2_O_2_ producing enzymes together with AA2 PODs and AA9 LPMOs has been demonstrated on wood substrates for many white rot fungi of *Agaricomycetes* (Vanden Wymelenberg et al., 2009, 2011; Hori et al., 2014; Alfaro et al., 2016; Kuuskeri et al., 2016), which confirms the synergism and necessity of these oxidoreductases for effective biodegradation of all wood lignocellulose components including lignin (Figure 1).

#### SOD Activity in Saprobic Basidiomycota

In this sense, SOD as hydrogen peroxide producing enzyme would logically enhance the extracellular oxidative chemistry and peroxidase activities. SOD has been associated with LPMO enzymes where the role of SOD has been determined as provider of H_2_O_2_ for enzyme activation and peroxygenation (Bissaro et al., 2020).

In the *Basidiomycota* white rot fungus *P. chrysosporium*, SOD activity has been addressed to the mitochondrial MnSOD1 (Belinky et al., 2002). In the study, *P. chrysosporium* indicated Mn-SOD activity while *Ascomycota* fungi also had Cu/Zn-SOD activity. In addition, expression of this Mn-SOD during initial phase of hyphal growth in *P. chrysosporium* was at the highest level while in species of *Ascomycota*, SOD production increased during stationary phase of fungal growth (Belinky et al., 2002). In another study, expression of SOD was induced in *P. chrysosporium* as an antioxidant response to environmental pollutants such as silver nanoparticles (Huang et al., 2018).

According to our bioinformatic search, the genome of *P. chrysosporium* harnesses 4 genes for SOD enzymes: one for Cu/Zn-SOD (SOD1 type) and 3 for Mn-SOD (SOD2/3 types), which is a typical set of SOD encoding genes in the *Basidiomycota* order *Polyporales* (Figure 2B, Supplementary Table 1). Previously, it was noticed that in species of white rot fungi, SOD activity followed formation of mycelial superoxide under temperature stress conditions (Fink-Boots et al., 1999). In this respect, it may be assumed that different SOD-encoding genes respond to different abiotic stress factors—not only following ROS levels—as well as to endogenous regulation in the wood decay fungi.

Analogous to the endogenous regulation of Mn-SOD in *P. chrysosporium* (Belinky et al., 2002), expression and activity of mitochondrial Mn-SOD in the white rot fungus *Pleurotus ostreatus* of the order *Agaricales* was dependent on fungal life cycle and growth stage: gene expression and SOD enzyme activity were higher in fruiting bodies (basidiocarps, mushrooms) in contrast to the vegetative mycelia or primordial stage (Yin C. et al., 2015). Changes in expression of Mn-SOD and enzyme activity were suggested to be associated with environmental stress factors influencing the developmental stages (Yin C. et al., 2015).

SOD activity apparently has a critical role in determining the shelf-life of mushrooms of edible fungi of *Basidiomycota* (Dama et al., 2010). In *Pleurotus eryngii*, high CO_2_ and low O_2_ atmosphere cause similar effect on mushroom shelf-life through SOD activity (Li et al., 2013). Moreover, alterations in the abundance of SOD and other antioxidant enzymes have been noticed in mycelial interactions of collaborative cultivations of white rot fungi (Zhong et al., 2018). In *Pleurotus pulmonarius*, production of activities of peroxidases involved in lignocellulose degradation were concomitant with generation of extracellular concentrations of H_2_O_2_ (Corrêa et al., 2016). Interestingly, intracellular SOD activity was high at the same time points. This may indicate an overall protection from intracellular oxidative agents during accelerated production of ROS (especially H_2_O_2_) for extracellular activities in the plant biomass degrading fungi of *Basidiomycota*.

#### SOD Activity in Ectomycorrhizal Fungi

In symbiotic ectomycorrhizal (ECM) species of *Basidiomycota*, their tolerance to soil pollutants, particularly to harmful concentrations of metal ions, has yet a different type of relationship with ROS. Metal ions—mainly the alkali metal cations K^+^ and Na^+^ as well as Rb^+^ and Cs^+^–are known to generate superoxide in the presence of H_2_O_2_ in alkaline solutions, leading to formation of water and dissolved ${\text{O}}_{2}^{•-}$ (Zhdanov et al., 2005). Non-enzymatic manganese is known to protect against ${\text{O}}_{2}^{•-}$ in the cells (causing disproportionation of ${\text{O}}_{2}^{•-}$ into H_2_O_2_ and O_2_) (Archibald and Fridovich, 1981), especially as Mn phosphates and carbonates, whereas more tightly chelated Mn ions (as in citrates and pyrophosphates) do not have this ability (Barnese et al., 2012).

In soil environments, for instance cadmium pollutants are reported to increase SOD expression in microbes and fungi. SOD expression is generally induced in fungi as a response to heavy metal exposure. In detail, Cd induces production of H_2_O_2_ which apparently increases activities of SOD and CAT (Xu et al., 2015). ECM fungi of *Basidiomycota Agaricomycetes* extend their hyphae in soil and show species-specific responses and sensitivity to metal contaminants (Ott et al., 2002; Qi et al., 2016). SOD activity in the ECM fungi *Boletus edulis* and *Paxillus involutus* correlates with heavy metal stress (Ott et al., 2002; Collin-Hansen et al., 2005).

#### Basidiomycota and CAT Activity

In the white rot fungi *Pleurotus sajor caju* (order *Agaricales*) and *P. chrysosporium* (order *Polyporales*) CAT activity seems to follow production of superoxide during early degradation of sugarcane bagasse (Pompeu et al., 2019). However, after seven days of cultivation CAT activity decreased which led into lipid peroxidation as detected by generation of malondialdehyde. The authors suggested that high C:N ratio and ROS machinery may have caused these effects. Augmented temperatures lead to lipid peroxidation in *C. neoformans* (Brown et al., 2007). Therefore, endogenous ROS formation may lead to acceleration of lipid peroxidation. In these cases, the antioxidant activity of CAT most probably prevents harmful changes in the membrane structures (Ma et al., 2014).

Also, lower cultivation temperatures can indirectly increase oxidative stress and CAT activity in fungi. In *Polyporus umbellatus* formation of sclerotia was dependent on a shift to lower temperature, which promoted expression of NOX in concordance with increase in SOD and CAT activities (Xing et al., 2013). For instance, abiotic stress by drought and its damaging effects are countered by increase in CAT, SOD and extracellular POD activities in the wood-inhabiting fungus *Auricularia auricula-judae* (Ma et al., 2014).

Mycelial growth of the white rot species *Pycnoporus sanguineus* and *Trametes villosa* of *Polyporales* was diminished after exposure to plant extracts of *Casearia* spp. (Bento et al., 2014), which was explained by oxidative stress as detected in increasing CAT activity. In another study, it was concluded that species of *Pleurotus* have a higher ability to consume phenolics and flavonoids in comparison to the two tested *Ascomycota* species (*Aspergillus fumigatus* and *Paecilomyces variotii*) which exhibited higher CAT activity on fruit peel substrates (El-Katony et al., 2020). Secondary metabolism supposedly creates ROS which is countered by CAT also in the white rot fungus *P. chrysosporium* (Jiang et al., 2009).

Previously, it was reported that CAT enzyme has a minor role in stress response against ROS in *C. neoformans* (Brown et al., 2007). In addition, it was reported that catalases are not required for virulence of the fungus (Giles et al., 2005; Staerck et al., 2017). However, CAT together with SOD and thioredoxin reductase are considered as a part of the defense system in *C. neoformans* against the fungicide fluconazole (Peng et al., 2018). The latter is supported by our bioinformatic analyses finding five genes for CAT proteins in *C. neoformans* (Figure 2B, Supplementary Table 1).

## Conclusions

Our review includes a genomic survey of a multitude of ROS related intra- and extracellular enzymes among *Basidiomycota*, following taxonomic classification and focusing on different lifestyles between and within the systematic orders of fungi. In brief, it may be concluded that differences in the set of extracellular enzymes activated by ROS, especially by H_2_O_2_, and involved in generation of H_2_O_2_, follow the differences in fungal lifestyles. The wood and plant biomass degrading white rot fungi of *Agaricomycetes* contain the highest copy numbers for genes encoding various peroxidases, mono- and peroxygenases, and oxidases. However, there are some differences among the sets of intracellular thiol-mediation involving proteins, and existence of enzyme mechanisms for quenching of intracellular H_2_O_2_ and ROS. In animal and plant pathogen species, the extra- and intracellular peroxidases are seemingly in minor role than in independent saprobic, filamentous fungi of *Basidiomycota*. Regarding extracellular ROS enzymes, substantial reduction of several gene families differentiates the brown rot fungi, yeast type and pathogenic species from white rot, soft rot-other wood-decay type, litter-decomposing and symbiotic mycorrhizal *Basidiomycota*. Noteworthy is that our genomic survey and review of the literature point to that there are differences both in generation of extracellular ROS as well as in mechanisms of response to oxidative stress and mitigation of ROS between fungi of *Basidiomycota* and *Ascomycota*.

## Author Contributions

HM, JÖ-U, and TL designed the study. HM and JÖ-U performed the bioinformatic searches, data analyses, and illustrations. All authors participated in manuscript drafting and final editing was done by TL and JÖ-U. All authors contributed to the article and approved the submitted version.

## Funding

This study was supported by Jane and Aatos Erkko foundation (Grant Number: 170101 to TL).

## Conflict of Interest

The authors declare that the research was conducted in the absence of any commercial or financial relationships that could be construed as a potential conflict of interest. The handling editor LN declared a past collaboration with the authors TL.

## Publisher's Note

All claims expressed in this article are solely those of the authors and do not necessarily represent those of their affiliated organizations, or those of the publisher, the editors and the reviewers. Any product that may be evaluated in this article, or claim that may be made by its manufacturer, is not guaranteed or endorsed by the publisher.

## Supplementary Material

Supplementary Information Materials and methods.

Supplementary Table 1 Protein IDs per each fungal genome retrieved from MycoCosm with predicted protein functions, localizations, Blastp search identity %, of all candidate proteins included in the analyses. Second leaflet: list of reference protein model homologs used in the Blastp searches.

Supplementary Table 2 Information on the fungal species taxonomy and lifestyle, and genomes used in the genome mining.
