# Supplementary material for: Basidiomycota Fungi and ROS: Genomic Perspective on Key Enzymes Involved in Generation and Mitigation of Reactive Oxygen Species
Source: Front Fungal Biol. 2022 Mar 23;3:837605. doi: 10.3389/ffunb.2022.837605 (PMC10512322; doi:10.3389/ffunb.2022.837605)
Supplement: Supplementary Information — Materials and methods. [file Data_Sheet_1.PDF]

Supplementary Information. Materials and methods

Genome mining for proteins involved in ROS-related processes in *Basidiomycota* was performed based on 4393 protein models (Supplementary Table 1) from 67 representative genomes (Supplementary Table 2). These genomes were downloaded from JGI MycoCosm (http://mycocosm.jgi.doe.go) (Grigoriev et al. 2014) on November 4, 2020. A custom blast database was prepared from the protein models of each genome, and used as the reference database in Blastp searches (Altschul et al. 1990, Camacho et al. 2009). The following search criteria were used: -evalue 1e-5 -word_size 3 -matrix BLOSUM62 -seg yes. Well-characterized representative reference protein models (Supplementary Table 1, second leaflet) were used as query proteins.

After the Blastp search, most of the protein classifications, except for the GMC-AA3 proteins, were manually confirmed based on multiple sequence alignments using ClustalW (Thompson *et al*. 1994) including the functionally known reference proteins (Supplementary Table 1, second leaflet) as models, with default parameter settings, implemented in the BioEdit sequence alignment editor (Hall 1999) version 7.0.5.3. This was done because candidate protein homologs from the same protein family could not always be defined directly based on statistics from the Blastp analysis. Manual inspection of the amino-acid multiple sequence alignments helped in confirming the similarity of Blastp hits, detection of sequences with abnormal length/shortness or other aberrations, and rearrangement of similar proteins from protein families based on e.g. characteristic protein domains or catalytic sequences specific to certain enzymes. This way we could assure that proteins included in the count table were properly classified according to homology and contained essential domains and motifs.

On the other hand, some sequences lacking important motifs were included in the counts table (Figure 2A,B), if we were able to confirm that the gene model coding for the protein was mis-translated or -annotated (i.e. missing exon cases where the intron-exon junctions and correct coding sequence had been missed but was found in the gene model by inspection). The accepted protein models are listed in Supplementary Table 1, together with Blastp percent identity values. In some cases, the Blastp result for a certain protein was split into several Blastp hits, in which cases the percent identity value for the longest alignment is reported.

All candidate sequences were subjected to cellular localization prediction using DeepLoc 1.0 (Almagro Armenteros *et al*. 2017), with Profiles encoding.

Tailor-made identification processes for some protein families

For detection of putative cellulose-degrading CAZy AA9 LPMO encoding genes, Blastp searches using two query protein sequences (*Phlebia radiata* Phlrad1 annotated proteins 140653 and 145579, Mäkinen et al. 2019) were performed. The search results were combined and sequences less than 100 aa in length were discarded.

GMC oxidoreductase (CAZy AA3 enzymes) query sequences for Blastp searches were chosen according to a recent extensive study (Sützl et al. 2019) and are listed in Supplementary Table 1 (second leaflet). GOx was not included because it seems to be absent from *Basidiomycota* genomes (Sützl et al. 2019). Results from the 6 Blastp searches were combined, and each candidate sequence assigned to the protein class (AAO/PDH/AOx/CDH/GDH/Pox) for which it had obtained the lowest e-value. Subsequently, to obtain results comparable with the study, only sequences with a percent identity value greater than 35 were counted as real candidates. To identify the correct homologs of

CDH among the the Blastp result candidate proteins, Batch CD-Search (Marchler-Bauer & Bryant 2004, Marchler-Bauer *et al*. 2011) was applied to identify their Pfam domains, which for CDH are recognized as GMC_oxred_C ,GMC_oxred_N, CDH-cyt and sometimes also including CBM_1. The presence of a GMC dehydrogenase domain was set as a requirement which was met in only 46 candidate proteins.

For copper-radical oxidases (CRO, CAZy AA5 enzymes) Blastp search using the *P. chrysosporium* sequence for GLOX (UniProt Q01772, corresponding to MycoCosm Phchr2 protein ID 11068) identified proteins from all CRO subfamilies. In order to classify the Blastp hit candidates into the correct CRO subfamilies, multiple sequence alignment together with the characterised *P. chrysosporium* CRO protein sequences (Vanden Wymelenberg et al. 2006) was performed, and sequences rearranged according to similarity to the *P. chrysosporium* reference proteins (CRO1 Phchr2|2416765, CRO2 Phchr2|2970935. CRO3 Phchr2|1717398, CRO4 Phchr2|3032412, CRO5 Phchr2|3032414, CRO6 Phchr2|2894758).

Graphical representations of protein gene count data

The gene counts corresponding to the identified protein sequences were summarized and raw counts visualized as a heat map using Microsoft Excel. For Principal Components Analysis and visualization of the data as a bar plot, the gene count data was imported to R-studio for Windows (version 1.4.1106, with R version 4.1.0 (2021-05-18) -- "Camp Pontanezen") (R Core Team, 2021). Package tidyverse (Wickham et al. 2019) version 1.3.1 was used for data analysis. PCA was performed on the raw count table using the prcomp function (arguments center = TRUE, scale = FALSE) from the basic stats package. Results were plotted using the plot function in the graphics package, together with the RColorBrewer packade (Neuwirth 2014). Count representation as stacked bars was performed using functions of the ggplot2 (v. 3.3.3.) package in R environment.

References

Almagro Armenteros JJ, Sønderby CK, Sønderby SK, Nielsen H, Winther O (2017). DeepLoc: prediction of protein subcellular localization using deep learning. Bioinformatics 33(21):3387-3395. doi:10.1093/bioinformatics/btx431

Altschul SF, Gish W, Miller W, Myers EW, Lipman DJ (19990). Basic local alignment search tool. Journal of Molecular Biology 215(3):403-10. doi: 10.1016/S0022-2836(05)80360-2. PMID: 2231712. doi:10.1016/S0022-2836(05)80360-2

Camacho C, Coulouris G, Avagyan V, Ma N, Papadopoulos J, Bealer K, Madden TL (2009). BLAST+: architecture and applications. BMC Bioinformatics 10:421. doi: 10.1186/1471-2105-10-421. PMID: 20003500; PMCID: PMC2803857. doi.org/10.1186/1471-2105-10-421

Grigoriev IV, Nikitin R, Haridas S, Kuo A, Ohm R, Otillar R, Riley R, Salamov A, Zhao X, Korzeniewski F, Smirnova T, Nordberg H, Dubchak I, Shabalov I (2014). MycoCosm portal: gearing up for 1000 fungal genomes. Nucleic Acids Research 42(1):D699-704. doi:10.1093/nar/gkt1183

Hall TA (1999). BioEdit: a user-friendly biological sequence alignment editor and analysis program for Windows 95/98/NT. Nucleic Acids Symposium Series 41:95-98.

Mäkinen M, Kuuskeri J, Laine P, Smolander O-P, Kovalchuk A, Zeng Z, Asiegbu FO, Paulin L, Auvinen P, Lundell T (2019). Genome description of *Phlebia radiata* 79 with comparative genomics analysis on lignocellulose decomposition machinery of phlebioid fungi. BMC Genomics 20**,** 430. doi:10.1186/s12864-019-5817-8

Marchler-Bauer A, Bryant SH (2004). CD-Search: protein domain annotations on the fly. Nucleic Acids Research 32(suppl_2):W327–W331. doi:10.1093/nar/gkh454

Marchler-Bauer A, Lu S, Anderson J B, Chitsaz F, Derbyshire MK, DeWeese-Scott C, Fong JH, Geer LY, Geer RC, Gonzales NR, Gwadz M, Hurwitz DI, Jackson JD, Ke Z, Lanczycki CJ, Lu F, Marchler GH, Mullokandov M, Omelchenko MV, Robertson CL, Song JS, Thanki N, Yamashita RA, Zhang D, Zhang N, Zheng C, Bryant SH (2011). CDD: a Conserved Domain Database for the functional annotation of proteins. Nucleic acids research 39(Database issue):D225–D229. doi:10.1093/nar/gkq1189

Neuwirth E (2014). RColorBrewer: ColorBrewer Palettes. R package version 1.1-2. https://CRAN.R-project.org/package=RColorBrewer

Sützl L, Foley G, Gillam EMJ, Bodén M, Haltrich D (2019). The GMC superfamily of oxidoreductases revisited: analysis and evolution of fungal GMC oxidoreductases. Biotechnology for Biofuels 12**,** 118. doi:10.1186/s13068-019-1457-0

Sützl L, Laurent CVFP, Abrera AT, Schütz G, Ludwig R, Haltrich D (2018). Multiplicity of enzymatic functions in the CAZy AA3 family. Applied Microbiology and Biotechnology 102**:**2477–2492. doi:10.1007/s00253-018-8784-0

Thompson JD, Higgins DG, Gibson TJ (1994). CLUSTAL W: improving the sensitivity of progressive multiple sequence alignment through sequence weighting, position-specific gap penalties and weight matrix choice. Nucleic Acids Research 22(22):4673-80. doi:10.1093/nar/22.22.4673

Vanden Wymelenberg A, Sabat G, Mozuch M, Kersten PJ, Cullen D, Blanchette RA (2006). Structure, organization, and transcriptional regulation of a family of copper radical oxidase genes in the lignin-degrading basidiomycete *Phanerochaete chrysosporium*. Applied and Environmental Microbiology 72(7):4871-4877. doi:10.1128/AEM.00375-06

Wickham H, Averick M, Bryan J, Chang W, D'Agostino McGowan L, François R, Grolemund G, Hayes A, Henry L, Hester J, Kuhn M, Pedersen TL, Miller E, Bache SM, Müller K, Ooms J, Robinson D, Seidel DP, Spinu V, Takahashi K, Vaughan D, Wilke C, Woo K, Yutani H (2019). Welcome to the tidyverse. Journal of Open Source Software 4(43), 1686. doi:10.21105/joss.01686
